# Identification and Expression Analysis of G Protein-Coupled Receptors in the Miridae Insect *Apolygus lucorum*

**DOI:** 10.3389/fendo.2021.773669

**Published:** 2021-11-26

**Authors:** Han Gao, Yanxiao Li, Miao Wang, Xiaowen Song, Jing Tang, Fan Feng, Bin Li

**Affiliations:** Jiangsu Key Laboratory for Biodiversity and Biotechnology, College of Life Sciences, Nanjing Normal University, Nanjing, China

**Keywords:** identification, GPCRs, *Apolygus lucorum*, expansion, phylogenetic analysis

## Abstract

G protein-coupled receptors (GPCRs) are the largest and most versatile family of transmembrane receptors in the cell and they play a vital role in the regulation of multiple physiological processes. The family Miridae (Hemiptera: Heteroptera) is one of the most diverse families of insects. Until now, information on GPCRs has been lacking in Miridae. *Apolygus lucorum*, a representative species of the Miridae, is an omnivorous pest that occurs worldwide and is notorious for causing serious damage to various crops and substantial economic losses. By searching the genome, 133 GPCRs were identified in *A. lucorum*. Compared with other model insects, we have observed GPCR genes to be remarkably expanded in *A. lucorum*, especially focusing on biogenic amine receptors and neuropeptide receptors. Among these, there is a novel large clade duplicated from known FMRFamide receptors (FMRFaRs). Moreover, the temporal and spatial expression profiles of the 133 genes across developmental stages were determined by transcriptome analysis. Most GPCR genes showed a low expression level in the whole organism of *A. lucorum*. However, there were a few highly expressed GPCR genes. The highly expressed LW opsins in the head probably relate to nocturning of *A. lucorum*, and the expression of *Cirl* at different times and in different tissues indicated it may be involved in growth and development of *A. lucorum*. We also found C2 leucine-rich repeat-containing GPCRs (LGRs) were mainly distributed in Hemiptera and Phthiraptera among insects. Our study was the first investigation on GPCRs in *A. lucorum* and it provided a molecular target for the regulation and control of Miridae pests.

## 1 Introduction

G protein-coupled receptors (GPCRs) are in a large family of protein cell surface receptors that detect molecules outside the cell and activate cellular responses (1, 2). GPCRs are found only in eukaryotes, namely, yeast, choanoflagellates, and animals (3). Based on sequence homology and functional similarity, GPCRs can be grouped into six families (4, 5): Family-A (rhodopsin-like); Family-B (secretin receptor family); Family-C (metabotropic glutamate/pheromone); Family-D (fungal mating pheromone receptors); Family-E (cyclic AMP receptors); and Family-F (frizzled/smoothened). These receptors are involved in a wide variety of physiological processes (6), namely, visual sensation (7), taste (8), smell sensation (9), behavioral and mood regulation (10), regulation of immune system activity and inflammation (11, 12), and autonomic nervous system transmission (10, 13). Because of their crucial roles in the regulation of multiple physiological processes, GPCRs are an important drug target (14) and approximately 34% (15) of all Food and Drug Administration (FDA) approved drugs target 108 members of this family.

With the continuous innovation of next-generation sequencing technology and bioinformatics, systematic identification research about GPCRs has been reported in several insects (16–21). Among Hemiptera, it has been reported in *Acyrthosiphon pisum* (22), *Aphis craccivora* (23), *Cimex lectularius* (24), *Diaphorina citri* (25), *Nilaparvata lugens* (26), and *Rhodnius prolixus* (27). The family Miridae (Hemiptera: Heteroptera), which includes plant bugs, leaf bugs, or grass bugs, is one of the most diverse families of insects, including over 11,000 species in more than 1,300 genera (4, 28). It is the largest family of true bugs belonging to Hemiptera and new members of Miridae are being described constantly. Mirids exhibit a wide range of food preferences and behaviors, including phytophagy, carnivory, and omnivory. Some mirids exhibit significant economic impacts and some are pests of food and fiber crops, whereas others are beneficial species used as biological control agents (29). Although Miridae is the largest family of Hemiptera and exhibits a complex habit, there has been little information reported on GPCRs.

*Apolygus lucorum* (Miridae) is an omnivorous pest that occurs worldwide and is notorious for the serious damage it causes in various crops and its substantial economic losses (30, 31). Recently, the genome of *A. lucorum* had been reported, which provided convenient in-depth studies of this pest (32). In the present research, using bioinformatics analysis, we screened the genes encoding GPCRs from the genome of *A. lucorum*. The expression profiles of all GPCRs were also determined by using public transcriptome data. These results allowed us to make comparisons of GPCR systems in different insect species and to provide relevant information for further functional studies in *A. lucorum*. Our study was the first investigation of GPCRs in *A. lucorum*, which may become the basis for further investigation of the function of miridae GPCRs.

## 2 Materials and Methods

### 2.1 Identification of *A. lucorum* GPCRs

*A. lucorum* protein sequences were retrieved from the NCBI Genome database (https://www.ncbi.nlm.nih.gov/assembly/GCA_009739505.2/) (32). Based on previous studies and records in Flybase (http://www.flybase.org/) (33), the GPCRs of *Drosophila melanogaster* (34), *A. pisum* (22), *Bombyx mori* (17), *Tribolium castaneum* (16), and *Pediculus humanus* humanus (18) were collected. By using *D. melanogaster* GPCRs as references and *A. lucorum* protein sequences as queries, BLASTP searches (35) were performed with a cut-off e-value of 1e−5 to look for all GPCR candidates. Then, seven-transmembrane (7TM) domain and annotation information was adopted as the basic criteria for all GPCR candidates. The GPCR candidates in which the number of 7TM domains was more than four or the annotation information indicated it was a GPCR were retained. The remaining GPCR candidates were also confirmed by means of BLASTX analysis in the UniProtKB/Swiss-Prot database. Using all GPCRs that we collected, pre-phylogenetic analysis with the maximum likelihood method was the final criteria to remove non-GPCRs from candidate pools. If a candidate showed fewer genetic relationships with known GPCRs by phylogenetic analysis and the hit sequences in BLASTX analysis indicated they were not GPCRs, they were classified as a non-GPCR and removed from our analysis.

### 2.2 Structural Analyses, Annotation Information, and Gene Locations of GPCRs

The 7TM domains for all GPCR candidates were predicted with the server TMHMM (v2.0) (36) from the Centre for Biological Sequence Analysis (http://www.cbs.dtu.dk/services/TMHMM/). Functional annotations of the target proteins were done using InterProScan (37). In addition, the chromosomal location of each GPCR candidate was extracted from the genome annotation file of *A. lucorum*.

### 2.3 Phylogenetic Analysis

Partial GPCRs of *R. prolixus* and *C. lectularius* that also belonged to Heteroptera were also obtain based on previous study (24, 27). GPCRs from *D. melanogaster*, *A. pisum*, *C. lectularius*, and *R. prolixus* were assigned to a family/subfamily according to previous results (22, 24, 27, 34). Putative *A. lucorum* GPCRs were classified into different families/subfamilies according to the families to which their orthologous proteins were assigned. Amino acid sequences of the putative *A. lucorum* GPCRs in each family/subfamily were aligned with receptors of the same family/subfamily in *D. melanogaster*, *A. pisum*, *C. lectularius*, and *R. prolixus* using MAFFT v7 (38). Phylogeny tests were accomplished using the bootstrap method with 1,000 replications to reconstruct maximum likelihood (ML) trees using IQ-TREE (39) and the best-fit tree model was determined with ModelFinder (40). It should be noted that the GPCRs of *R. prolixus* and *C. lectularius* were uncompleted, which were composed of opsins, biogenic amine receptors, and neuropeptide GPCRs. For the *Drosophila* sequences, the name of the GPCRs were used, while for *A. pisum* and *R. prolixus*, the protein names were same as in previous work (22, 27), and for *C. lectularius*, the accession numbers in NCBI were used. The GPCRs of *A. lucorum* identified in this work were numbered according to their families.

### 2.4 Expression Analysis

To study the expression profiles of the GPCRs, a total of 39 transcriptome data of *A. lucorum* were downloaded from the genome project of *A. lucorum* (Accession: PRJNA526332) in the NCBI Sequence Read Archive (SRA) database (https://www.ncbi.nlm.nih.gov/sra/) (30, 41), which included egg and different tissues (leg, head, body, mouthpart, wing, and gut) of nymphs and adults. Each tissue contained three biological replicates. In detail, we downloaded the SRA data first and then we used an SRA-Toolkit to split the paired-end reads. Clean reads were obtained from the raw data using Trimmomatic (42) to remove reads with quality scores lower than 10 and adapter sequences. To analyze gene expression profiles, clean reads of each sample were mapped to *A. lucorum* gene sets using hisat2 (43), and then the TPM value (44) of each putative GPCR gene was calculated with featureCounts (45). These TPM expression values were scaled and served to generate a cross-sample normalized trimmed mean of the M-values (TMM) gene expression matrix (46). Finally, the heatmap was drawn in ITOL (https://itol.embl.de) (47) using the normalized matrix. The value used for each sample was the mean of three independent biological replicates.

### 2.5 Classification of Gene Duplication Types

MCScanX (48) was used to classified the duplication types of different duplicate GPCR genes. First, the homology with different genes in the genome of *A. lucorum* was determined by a whole-genome BLASTP analysis with a max target seqs of 5 and a cut-off e-value of 1e−5. Then, the homology with different genes and the chromosomal location were combined and all genes were classified into various types, including the segmental duplication, and tandem duplication. Finally, the duplication types of GPCRs were extracted based on these results. All visualized works were accomplished in TBtools (49).

## 3 Results

**Figure 1:**
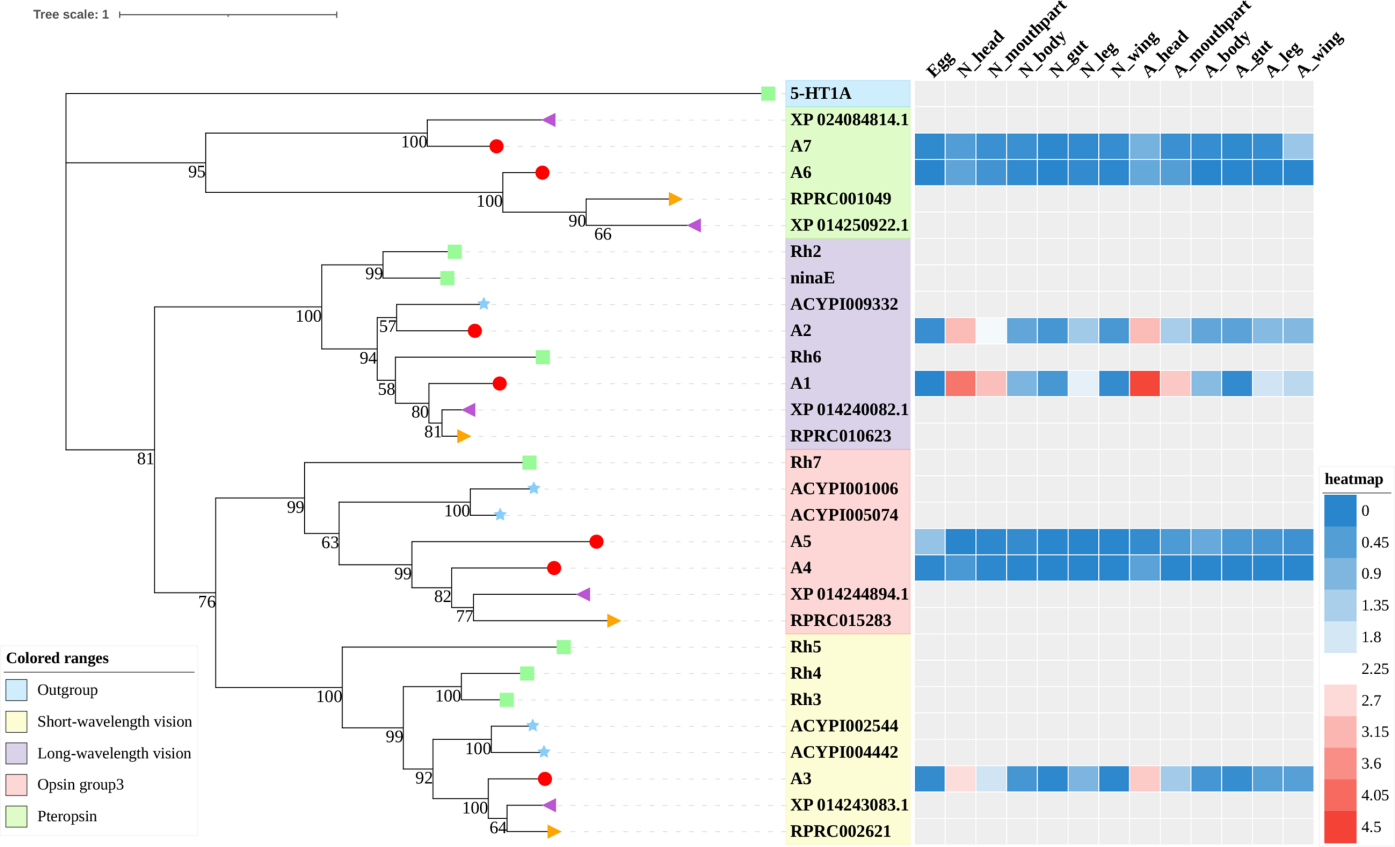
Phylogenetic tree reconstruction of opsin GPCRs from *D. melanogaster* (green square), *A. pisum* (blue star), *R. prolixus* (orange right triangle), *C. lectularius* (purple left triangle), and *A. lucorum* (red circle) inferred from maximum likelihood (ML) analysis. Numbers at nodes on the tree were the bootstrap values (below 50 are not shown). The tree was rooted by the *D. melanogaster* biogenic amine receptor 5-HT1A. Expression profiles of *A. lucorum* GPCR genes from different tissues are shown in the corresponding branch side. The transcription level of each gene is represented by a square with a color that codes for the values of Lg (TPM+1). N, nymph; A, adult.

**Figure 2:**
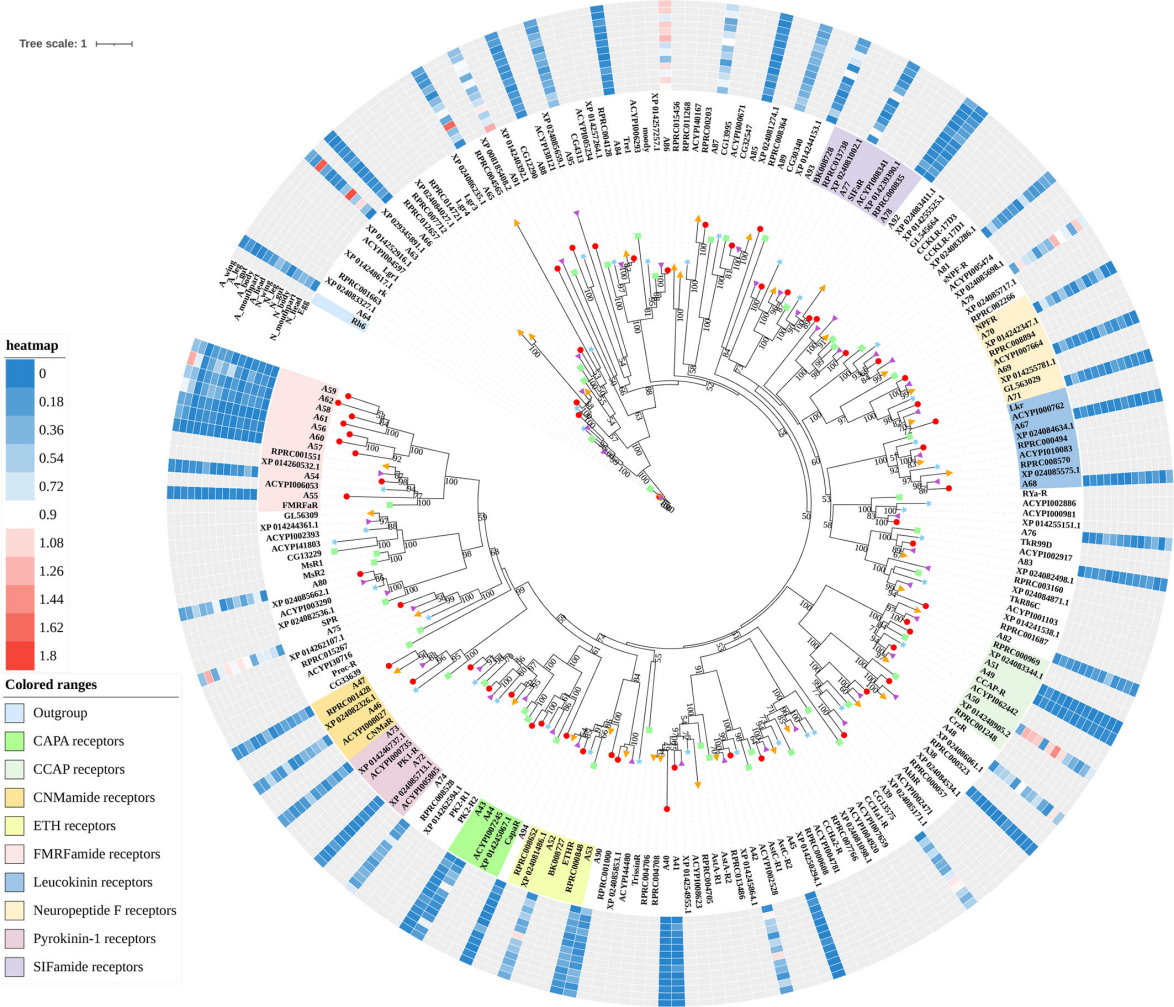
Phylogenetic tree reconstruction of neuropeptide and protein hormone receptors from *D. melanogaster* (green square), *A. pisum* (blue star), *R. prolixus* (orange right triangle), *C. lectularius* (purple left triangle), and *A. lucorum* (red circle) inferred from maximum likelihood (ML) analysis. Numbers at nodes on the tree were the bootstrap values (below 50 are not shown). The tree was rooted by the *D. melanogaster* opsin GPCR Rh6. Expression profiles of *A. lucorum* GPCR genes from different tissues are shown in the corresponding branch side. The transcription level of each gene is represented by a square with a color that codes for the values of Lg (TPM+1). N, nymph; A, adult.

**Figure 3:**
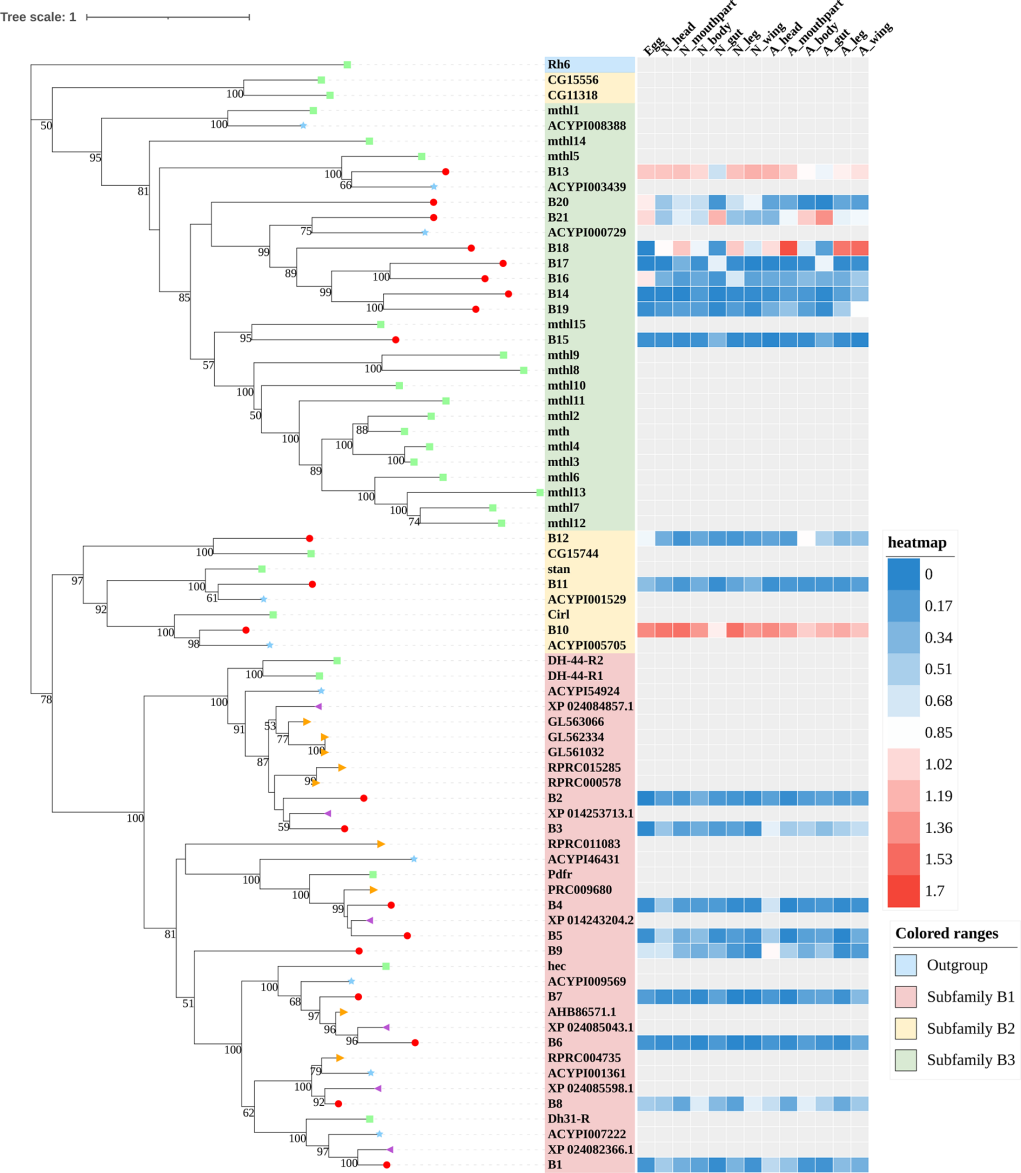
Phylogenetic tree reconstruction of Family-B GPCRs from *D. melanogaster* (green square), *A. pisum* (blue star), *R. prolixus* (orange right triangle), *C. lectularius* (purple left triangle), and *A. lucorum* (red circle) inferred from maximum likelihood (ML) analysis. Numbers at nodes on the tree were the bootstrap values (below 50 are not shown). The tree was rooted by the *D. melanogaster* opsin GPCR Rh6. Expression profiles of *A. lucorum* GPCR genes from different tissues are shown in the corresponding branch side. The transcription level of each gene is represented by a square with a color that codes for the values of Lg (TPM+1). N, nymph; A, adult.

**Figure 4:**
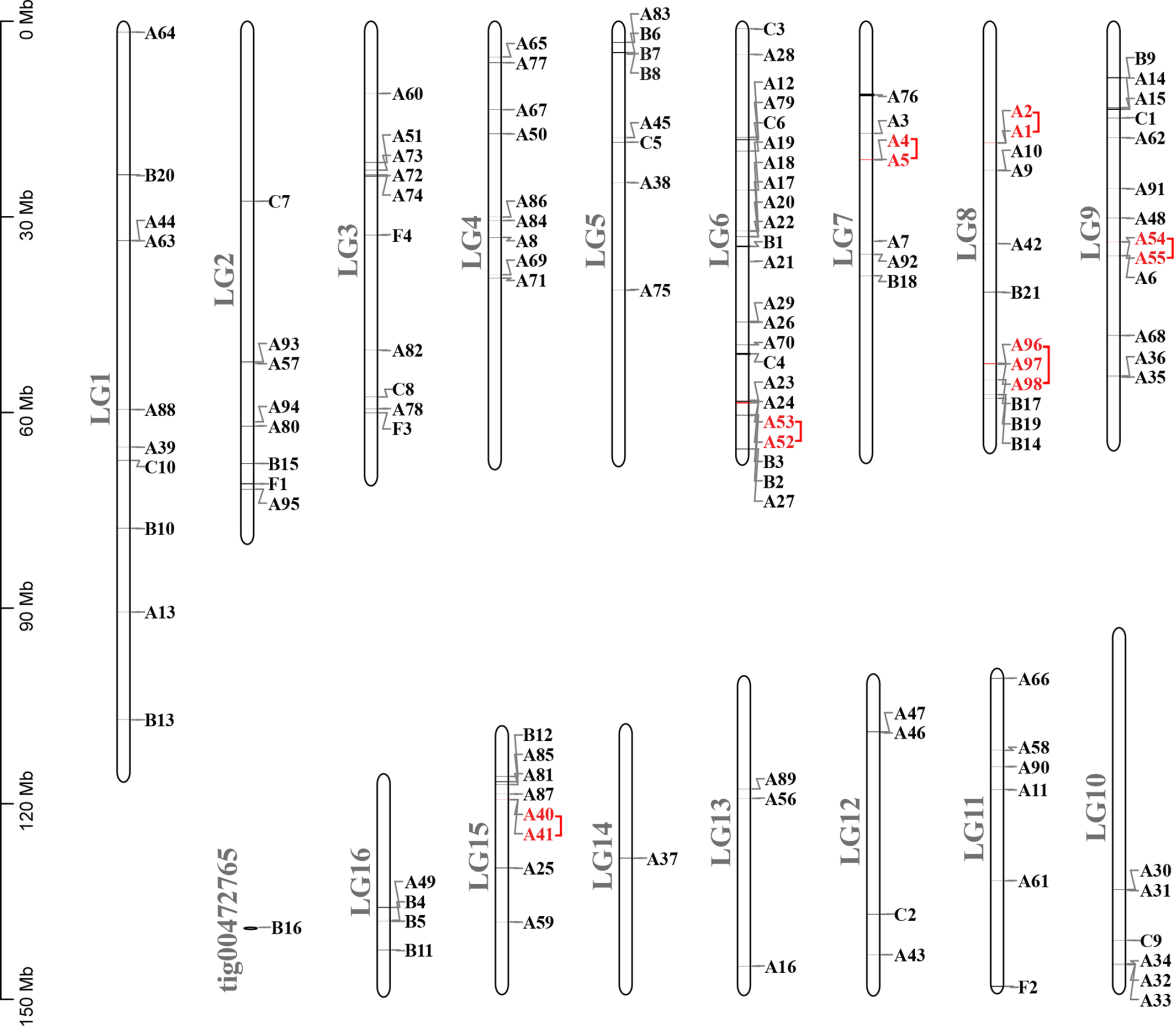
Chromosomal locations and tandem duplicated gene pairs of the 133 putative GPCR genes. Each was mapped to the chromosome based on its physical location. The chromosome number (LG1–LG16, tig00472765) is indicated at the left. The tandem duplicated genes were outlined with red color.

A total of 133 putative GPCRs were identified in *A. lucorum*. These GPCRs were classified into four families and included 98 family-A members, 21 family-B members, 10 family-C members, and four family-F members (**Tables 1**–**4** and **Table S1**). Based on the protein sequences, phylogenetic trees were reconstructed for each GPCR family/subfamily of *A. lucorum*, *R. prolixus*, *A. pisum*, and *D. melanogaster*. All GPCRs were quantified with the TPM values obtained from transcriptomic data. The expression profile of each GPCR across developmental stages was also present in the phylogenetic trees of each GPCR family/subfamily (**Figures 1**–**3**, and **Figures S1–S3**). The chromosomal locations of all GPCRs are shown in **Figure 4**.

**Table 1:** Opsin and biogenic amine receptors in *A. lucorum*.

No.	Accession number	Putative Endogenous ligand	Orthologue of *D. melanogaster*	Orthologue of *A. pisum*	Predicted TMHs	Annotation by InterProScan	Homology search in Swissport (blastp)		
							E-value	Description	Species
**Opsin**									
A1	KAF6206346.1	Orphan	Rh6	ACYPI009332	Complete	(IPR000276) GPCR, rhodopsin-like; (IPR001760) Opsin; (IPR001391) Opsin lateral eye type	0	Opsin-1	*Schistocerca gregaria*
A2	KAF6206345.1	Orphan	Rh6	ACYPI009332	Complete	(IPR000276) GPCR, rhodopsin-like; (IPR001760) Opsin; (IPR001391) Opsin lateral eye type;	0	Opsin	*Sphodromantis* sp.
A3	KAF6207755.1	Orphan	Rh3, Rh4	ACYPI002544, ACYPI004442	Complete	(IPR000276) GPCR, rhodopsin-like; (IPR001760) Opsin; (IPR000856) Opsin RH3/RH4	1.00E−165	UV-sensitive opsin	*Apis mellifera*
A4	KAF6207831.1	Orphan	Rh7	ACYPI001006, ACYPI005074	Complete	(IPR000276) GPCR, rhodopsin-like	1.60E−77	Opsin-2	*Manduca sexta*
A5	KAF6207832.1	Orphan	Rh7	ACYPI001006, ACYPI005074	Complete	(IPR000276) GPCR, rhodopsin-like; (IPR001760) Opsin	1.88E−73	Opsin Rh3	*D. melanogaster*
A6	KAF6205310.1	Orphan	na	na	5	(IPR000276) GPCR, rhodopsin-like	4.83E−48	Pinopsin	*Columba livia*
A7	KAF6208054.1	Orphan	na	na	6	(IPR000276) GPCR, rhodopsin-like; (IPR001760) Opsin	2.01E−70	GQ-rhodopsin	*Mizuhopecten yessoensis*
**Biogenic amine receptors**									
A8	KAF6211999.1	Acetylcholine	mAChR-A	ACYPI005180	Complete	(IPR000276) GPCR, rhodopsin-like; (IPR000995) Muscarinic acetylcholine receptor family	4.10E−160	mAChR DM1	*D. melanogaster*
A9	KAF6206451.1	Acetylcholine	mAChR-B	ACYPI001255	6	(IPR000276) GPCR, rhodopsin-like	2.84E−57	mAChR gar-2	*Caenorhabditis elegans*
A10	KAF6206450.1	Acetylcholine	mAChR-B	ACYPI001255	2	(IPR000276) GPCR, rhodopsin-like	1.28E−27	mAChR gar-2	*Caenorhabditis elegans*
A11	KAF6202800.1	Acetylcholine	mAChR-C	na	Complete	(IPR000276) GPCR, rhodopsin-like	1.07E−23	D(1B) DopR	*Rattus norvegicus*
A12	KAF6209068.1	Dopamine	Dop1R1	ACYPI006935	Complete	(IPR000276) GPCR, rhodopsin-like; (IPR000929) Dopamine receptor family	0	Dop1R1	*D. melanogaster*
A13	KAF6217029.1	Dopamine	Dop1R2	ACYPI009241	6	(IPR000276) GPCR, rhodopsin-like	4.60E−164	Dop1R2	*D. melanogaster*
A14	KAF6204820.1	Dopamine	Dop2R	ACYPI007415	2	(IPR000276) GPCR, rhodopsin-like; (IPR001671) Melanocortin/ACTH receptor	2.03E−61	Dop2R	*D. melanogaster*
A15	KAF6204823.1	Dopamine	Dop2R	ACYPI007415	2	(IPR000276) GPCR, rhodopsin-like	2.20E−103	Dop2R	*D. melanogaster*
A16	KAF6201362.1	Dopamine, Ecdysteroids	DopEcR	ACYPI005538	Complete	(IPR000276) GPCR, rhodopsin-like	5.89E−24	G-protein coupled receptor 52	*Mus musculus*
A17	KAF6209377.1	Octopamine	Oamb	ACYPI005578	5	(IPR000276) GPCR, rhodopsin-like	5.20E−109	Oamb	*D. melanogaster*
A18	KAF6209376.1	Octopamine	Oamb	ACYPI005578	3	(IPR000276) GPCR, rhodopsin-like	2.03E−29	Oamb	*D. melanogaster*
A19	KAF6209232.1	Octopamine	Octbeta1R	ACYPI007386	5	(IPR000276) GPCR, rhodopsin-like	2.90E−104	Octbeta1R	*D. melanogaster*
A20	KAF6209392.1	Octopamine	Octbeta2R	ACYPI004658	3	(IPR000276) GPCR, rhodopsin-like	4.18E−91	Octbeta2R	*D. melanogaster*
A21	KAF6209465.1	Octopamine	Octalpha2R	ACYPI010155	4	(IPR000276) GPCR, rhodopsin-like	4.37E−54	Alpha-2C adrenoreceptor	*Danio rerio*
A22	KAF6209394.1	Octopamine	Octbeta2R	ACYPI004658	4	(IPR000276) GPCR, rhodopsin-like	6.10E−76	Octbeta2R	*D. melanogaster*
A23	KAF6209913.1	Octopamine	Octbeta3R	ACYPI010025	5	(IPR000276) GPCR, rhodopsin-like	6.00E−100	Octbeta3R	*D. melanogaster*
A24	KAF6209915.1	Octopamine	Octbeta3R	ACYPI010025	3	(IPR000276) GPCR, rhodopsin-like	2.09E−44	Octbeta3R	*D. melanogaster*
A25	KAF6199206.1	Octopamine/Tyramine	Oct-TyrR	ACYPI007379	Complete	(IPR000276) GPCR, rhodopsin-like; (IPR002002) Octopamine receptor	0	OctR	*Heliothis virescens*
A26	KAF6209650.1	Serotonin	5-HT1A, 5-HT1B	XP_001949725	4	(IPR000276) GPCR, rhodopsin-like	4.80E−107	5-HT receptor	*Bombyx mori*
A27	KAF6210093.1	Serotonin	5-HT1A, 5-HT1B	XP_001949725	4	(IPR000276) GPCR, rhodopsin-like	8.60E−103	5-HT receptor	*Heliothis virescens*
A28	KAF6208815.1	Serotonin	5-HT1A, 5-HT1B	XP_001949725	2	(IPR000276) GPCR, rhodopsin-like	1.71E−51	5-HT receptor	*Heliothis virescens*
A29	KAF6209646.1	Serotonin	5-HT1A, 5-HT1B	XP_001949725	2	(IPR000276) GPCR, rhodopsin-like	1.06E−41	5-HT receptor	*Bombyx mori*
A30	KAF6204179.1	Serotonin	5-HT2A	ACYPI008969	2	(IPR000276) GPCR, rhodopsin-like	5.75E−29	5-HT-2C	*Canis lupus familiaris*
A31	KAF6204181.1	Serotonin	5-HT2A	ACYPI008969	2	(IPR000276) GPCR, rhodopsin-like	4.89E−25	5-HT-2B	*Mus musculus*
A32	KAF6204453.1	Serotonin	5-HT2B	ACYPI50707	3	(IPR000276) GPCR, rhodopsin-like	2.15E−19	5-HT-2B	*Homo sapiens*
A33	KAF6204455.1	Serotonin	5-HT2B	ACYPI50707	3	(IPR000276) GPCR, rhodopsin-like	9.03E−24	5-HT-2B	*Mus musculus*
A34	KAF6204452.1	Orphan	na	na	Complete	(IPR000276) GPCR, rhodopsin-like	3.57E−14	5-HT-2A	*Macaca mulatta*
A35	KAF6205718.1	Serotonin	5-HT7	XP_003241835	5	(IPR000276) GPCR, rhodopsin-like	8.50E−112	5-HT receptor	*D. melanogaster*
A36	KAF6205714.1	Serotonin	5-HT7	XP_003241835	2	(IPR000276) GPCR, rhodopsin-like	5.89E−53	5-HT receptor	*D. melanogaster*
A37	KAF6200010.1	Orphan	CG13579	ACYPI008777	5	(IPR000276) GPCR, rhodopsin-like	3.35E−12	Trace amine-associated receptor 1	*Mus musculus*

na, not annotated or not applicable, Complete means there is a complete 7TM structure.

**Table 2:** Neuropeptide and protein hormone receptors and purine GPCRs in *A. lucorum*.

No.	Accession number	Putative endogenous ligand	Orthologue of *D. melanogaster*	Orthologue of *A. pisum*	Predicted TMHs	Annotation by InterProScan	Homology search in Swissport (blastp)		
							E-value	Description	Species
**Neuropeptide and protein hormone receptors**									
A38	KAF6210560.1	AKH/corazonin-related peptide	na	na	4	(IPR000276) GPCR, rhodopsin-like; (IPR000405) Galanin receptor family	4.74E−31	GnRHR II	*Clarias gariepinus*
A39	KAF6216586.1	Adipokinetic hormone	AkhR	ACYPI002471	Complete	(IPR027417) P-loop containing nucleoside triphosphate hydrolase;	5.09E−49	GnRHR II	*Clarias gariepinus*
A40	KAF6198962.1	Allatostatins-A	AstA-R1, AstA-R2	ACYPI008623	4	None predicted	5.02E−41	AstA-R	*Bombyx mori*
A41	KAF6198963.1	Allatostatins-A	AstA-R1, AstA-R2	ACYPI008623	4	(IPR000276) GPCR, rhodopsin-like; (IPR005390) Neuromedin U receptor	2.45E−79	AstA-R	*Bombyx mori*
A42	KAF6206708.1	Allatostatins-C	AstC-R1, AstC-R2	ACYPI002528	Complete	(IPR000276) GPCR, rhodopsin-like; (IPR002131) Glycoprotein hormone receptor family; (IPR036055) LDL receptor-like superfamily; (IPR032675) Leucine-rich repeat domain superfamily	1.27E−81	Somatostatin receptor type 4	*Rattus norvegicus*
A43	KAF6202370.1	CAPA	CapaR	ACYPI007245	Complete	(IPR000276) GPCR, rhodopsin-like	1.17E−85	CapaR	*D. melanogaster*
A44	KAF6216164.1	CAPA	CapaR	ACYPI007245	Complete	(IPR000276) GPCR, rhodopsin-like; (IPR019427) 7TM GPCR, serpentine receptor class w (Srw)	6.22E−102	Cap2bR	*D. melanogaster*
A45	KAF6210431.1	CCHamide	CCHa2-R	ACYPI004781	Complete	(IPR000276) GPCR, rhodopsin-like	7.23E−131	CCHa1-R	*D. melanogaster*
A46	KAF6201633.1	CNMamide	CNMaR	ACYPI008027	2	(IPR000276) GPCR, rhodopsin-like; (IPR000611) Neuropeptide Y receptor family	NA	NA	NA
A47	KAF6201631.1	CNMamide	CNMaR	ACYPI008027	Complete	(IPR000276) GPCR, rhodopsin-like	9.79E−14	FMRFaR	*D. melanogaster*
A48	KAF6205178.1	Corazonin	CrzR	ACYPI002471	Complete	(IPR000276) GPCR, rhodopsin-like	3.56E−59	GnRHR	*Octopus vulgaris*
A49	KAF6198396.1	Crustacean cardioactive peptide	CCAP-R	ACYPI062442	Complete	(IPR000276) GPCR, rhodopsin-like; (IPR001634) Adenosine receptor	3.69E−158	CCAPR	*D. melanogaster*
A50	KAF6211684.1	Crustacean cardioactive peptide	CCAP-R	ACYPI062442	6	(IPR000276) GPCR, rhodopsin-like	1.9E−156	CCAPR	*D. melanogaster*
A51	KAF6213085.1	Crustacean cardioactive peptide	CCAP-R	ACYPI062442	Complete	(IPR008429) Cleft lip and palate transmembrane 1	1.73E−137	CCAPR	*D. melanogaster*
A52	KAF6209917.1	ETH	ETHR	BK008727	Complete	(IPR000276) GPCR, rhodopsin-like; (IPR000611) Neuropeptide Y receptor family	2.19E−44	TRH-R	*Gallus gallus*
A53	KAF6209916.1	ETH	ETHR	BK008727	3	(IPR000276) GPCR, rhodopsin-like; (IPR000611) Neuropeptide Y receptor family; (IPR036241) NSFL1 cofactor p47, SEP domain superfamily	4.21E−16	TRH-R	*Bos taurus*
A54	KAF6209916.1	FMRFamides	FMRFaR	ACYPI006053	Complete	(IPR000276) GPCR, rhodopsin-like; (IPR000611) Neuropeptide Y receptor family	1.75E−130	FMRFaR	*D. melanogaster*
A55	KAF6205268.1	FMRFamides	FMRFaR	ACYPI006053	5	(IPR027417) P-loop containing nucleoside triphosphate hydrolase; (IPR042035) DEAH helicase, winged-helix domain; (IPR012340) Nucleic acid-binding, OB-fold	5.96E−45	FMRFaR	*D. melanogaster*
A56	KAF6200840.1	FaRP	na	na	6	(IPR000276) GPCR, rhodopsin-like; (IPR019427) 7TM GPCR, serpentine receptor class w (Srw)	4.53E−40	FMRFaR	*D. melanogaster*
A57	KAF6215133.1	FaRP	na	na	5	(IPR000276) GPCR, rhodopsin-like	5.5E−45	FMRFaR	*D. melanogaster*
A58	KAF6202705.1	FaRP	na	na	Complete	(IPR000276) GPCR, rhodopsin-like; (IPR032675) Leucine-rich repeat domain superfamily; (IPR008112) Relaxin receptor	2.25E−31	FMRFaR	*D. melanogaster*
A59	KAF6199396.1	FaRP	na	na	5	(IPR000276) GPCR, rhodopsin-like	9.69E−35	FMRFaR	*D. melanogaster*
A60	KAF6212897.1	FaRP	na	na	Complete	(IPR000276) GPCR, rhodopsin-like	1.13E−33	FMRFaR	*D. melanogaster*
A61	KAF6203114.1	FaRP	na	na	6	(IPR000276) GPCR, rhodopsin-like	3.58E−26	FMRFaR	*D. melanogaster*
A62	KAF6204912.1	FaRP	na	na	Complete	(IPR000276) GPCR, rhodopsin-like	2.55E−29	FMRFaR	*D. melanogaster*
A63	KAF6216165.1	GPA2/GPB5	Lgr1	ACYPI004597	Complete	(IPR000276) GPCR, rhodopsin-like	2.6E−103	LH/CG-R	*Mus musculus*
A64	KAF6215698.1	Bursicon	rk	ACYPI000221	Complete	(IPR008365) Prostanoid receptor	7.46E−112	LGR5	*Rattus norvegicus*
A65	KAF6211436.1	Insulin-like peptide 7 and 8	Lgr3	ACYPI008291	5	(IPR000276) GPCR, rhodopsin-like	2.26E−116	Relaxin receptor 2	*Mus musculus*
A66	KAF6202512.1	Insulin-like peptide 7 and 8	Lgr4	na	Complete	(IPR000276) GPCR, rhodopsin-like	0	GPCR GRL101	*Lymnaea stagnalis*
A67	KAF6211616.1	Leucokinin	Lkr	ACYPI010083, ACYPI000762	5	(IPR000276) GPCR, rhodopsin-like; (IPR001681) Neurokinin receptor	2.25E−40	TkR99D	*D. melanogaster*
A68	KAF6205586.1	Leucokinin	Lkr	ACYPI010083, ACYPI000762	5	(IPR000276) GPCR, rhodopsin-like	5.04E−34	QRFP-like peptide receptor	*Branchiostoma floridae*
A69	KAF6212176.1	Neuropeptide F	NPFR	ACYPI007664	4	(IPR000276) GPCR, rhodopsin-like	1.66E−25	NPYR type 2	*Cavia porcellus*
A70	KAF6209720.1	Neuropeptide F	NPFR	ACYPI007664	4	(IPR000276) GPCR, rhodopsin-like; (IPR000611) Neuropeptide Y receptor family	4.36E−27	DmNPFR1	*D. melanogaster*
A71	KAF6212186.1	Neuropeptide F	NPFR	ACYPI007664	2	(IPR000276) GPCR, rhodopsin-like; (IPR000611) Neuropeptide Y receptor family	8.96E−16	NPFR	*D. melanogaster*
A72	KAF6213141.1	Pyrokinin-1	PK1-R	ACYPI000735, ACYPI005805	Complete	(IPR000276) GPCR, rhodopsin-like; (IPR002120) Thyrotropin-releasing hormone receptor	4.33E−117	PK1-R	*D. melanogaster*
A73	KAF6213123.1	Pyrokinin-1	PK1-R	ACYPI000735, ACYPI005805	3	(IPR000276) GPCR, rhodopsin-like; (IPR000611) Neuropeptide Y receptor family	2.91E−42	PK1-R	*D. melanogaster*
A74	KAF6213143.1	Pyrokinin-2	PK2-R2	na	Complete	(IPR000276) GPCR, rhodopsin-like; (IPR019427) 7TM GPCR, serpentine receptor class w (Srw)	1.14E−101	PK1-R	*D. melanogaster*
A75	KAF6210859.1	Proctolin	Proc-R	ACYPI30716	Complete	(IPR027417) P-loop containing nucleoside triphosphate hydrolase	3.98E−25	FMRFaR	*D. melanogaster*
A76	KAF6207650.1	RYamide	RYa-R	ACYPI002886	Complete	(IPR000832) GPCR, family 2, secretin-like	1.52E−122	RYa-R	*D. melanogaster*
A77	KAF6211457.1	SIFamide	SIFaR	ACYPI008341, BK008728	Complete	(IPR000276) GPCR, rhodopsin-like; (IPR002131) Glycoprotein hormone receptor family; (IPR032675) Leucine-rich repeat domain superfamily	8.27E−107	SIFaR	*D. melanogaster*
A78	KAF6213872.1	SIFamide	SIFaR	ACYPI008341, BK008728	3	(IPR008429) Cleft lip and palate transmembrane 1; (IPR030434) Cleft lip and palate transmembrane protein 1-like protein	4.69E−101	SIFaR	*D. melanogaster*
A79	KAF6209074.1	short neuropeptide F	sNPF-R	ACYPI005474	Complete	(IPR000276) GPCR, rhodopsin-like; (IPR005390) Neuromedin U receptor	1.15E−62	NPY2-R	*Homo sapiens*
A80	KAF6215350.1	Allatostatin-C	AstC-R	ACYPI003290	Complete	(IPR000276) GPCR, rhodopsin-like; (IPR032675) Leucine-rich repeat domain superfamily; (IPR002131) Glycoprotein hormone receptor family	3.65E−147	SPR	*D. melanogaster*
A81	KAF6198907.1	Sulfakinin	CCKLR-17D1, CCKLR-17D3	na	3	(IPR000276) GPCR, rhodopsin-like	3.51E−29	CCK-XLR	*Xenopus laevis*
A82	KAF6213654.1	Tachykinin	TkR86C	ACYPI001103	3	(IPR000276) GPCR, rhodopsin-like	4.57E−50	TkR86C	*D. melanogaster*
A83	KAF6210188.1	Tachykinin	TkR99D	ACYPI002917	4	(IPR000276) GPCR, rhodopsin-like	2.1E−93	TkR99D	*D. melanogaster*
A84	KAF6211953.1	Orphan	CG4313	ACYPI005234	5	(IPR000276) GPCR, rhodopsin-like	2.38E−55	moody	*D. melanogaster*
A85	KAF6198896.1	Orphan	CG32547	ACYPI000671	3	(IPR000276) GPCR, rhodopsin-like; (IPR001817) Vasopressin receptor	NA	NA	*NA*
A86	KAF6211940.1	Orphan	moody	ACYPI006293	6	(IPR000276) GPCR, rhodopsin-like	5.19E−143	moody	*D. melanogaster*
A87	KAF6198937.1	Orphan	na	ACYPI40167	Complete	(IPR000276) GPCR, rhodopsin-like	1.52E−14	NPY2-R	*Mus musculus*
A88	KAF6216499.1	Orphan	na	ACYPI38121	Complete	(IPR000276) GPCR, rhodopsin-like	1.14E−10	Melatonin receptor type 1A	*Gallus gallus*
A89	KAF6200805.1	Orphan	na	na	6	(IPR000276) GPCR, rhodopsin-like	1.02E−12	Melatonin receptor type 1A	*Gallus gallus*
A90	KAF6202756.1	Orphan	na	na	2	(IPR000276) GPCR, rhodopsin-like	1.17E−13	Somatostatin receptor type 5	*Homo sapiens*
A91	KAF6205087.1	Orphan	na	na	Complete	(IPR000276) GPCR, rhodopsin-like	5.87E−12	Prostaglandin E2 receptor EP4 subtype	*Bos taurus*
A92	KAF6208108.1	Orphan	na	na	Complete	(IPR000276) GPCR, rhodopsin-like; (IPR001556) Bombesin receptor-like	1.75E−41	RYa-R	*D. melanogaster*
A93	KAF6215130.1	Allatotropin	na	na	Complete	(IPR000276) GPCR, rhodopsin-like; (IPR005390) Neuromedin U receptor	8.144E−89	Orexin receptor type 2	*Mus musculus*
A94	KAF6215334.1	Orphan	na	na	5	(IPR000276) GPCR, rhodopsin-like	1.53E−53	TRHR	*Gallus gallus*
A95	KAF6215529.1	Orphan	na	na	Complete	(IPR000276) GPCR, rhodopsin-like	2.03E−13	Cadherin EGF LAG seven-pass G-type receptor 1	*Mus musculus*
**Purine receptor**									
A96	KAF6207107.1	Adenosine	AdoR	ACYPI24713	3	(IPR000276) GPCR, rhodopsin-like; (IPR001817) Vasopressin receptor	1.34E−29	AdoR A2a	*Equus caballus*
A97	KAF6207108.1	Adenosine	AdoR	ACYPI24713	6	None predicted	1.46E−57	AdoR A2a	*Equus caballus*
A98	KAF6207109.1	Adenosine	AdoR	ACYPI24713	3	(IPR000276) GPCR, rhodopsin-like	4.05E−21	AdoR A2a	*Gallus gallus*

na, not annotated or applicable, Complete means there is a complete 7TM structure.

**Table 3:** Family-B GPCRs of *A. lucorum*.

No.	Accession number	Putative endogenous ligand	Orthologue of *D. melanogaster*	Orthologue of *A. pisum*	Predicted TMHs	Annotation by InterProScan	Homology search in Swissport (blastp)		
							E-value	Description	Species
**SUBFAMILY B1**									
B1	KAF6209407.1	Diuretic hormone 31	Dh31-R	ACYPI007222, ACYPI001361	Complete	(IPR000832) GPCR, family 2, secretin-like; (IPR036445) GPCR family 2, extracellular hormone receptor domain superfamily	4.8E−74	Calcitonin gene-related peptide type 1 receptor	*Danio rerio*
B2	KAF6209957.1	Diuretic hormone 44	Dh44-R1, Dh44-R2	ACYPI54924	5	(IPR000832) GPCR, family 2, secretin-like; (IPR036445) GPCR family 2, extracellular hormone receptor domain superfamily; (IPR002001) GPCR, family 2, diuretic hormone receptor	5.79E−68	DH-R	*Acheta domesticus*
B3	KAF6209955.1	Diuretic hormone 44	Dh44-R1, Dh44-R2	ACYPI54924	2	(IPR000832) GPCR, family 2, secretin-like	5.51E−46	DH-R	*Acheta domesticus*
B4	KAF6198455.1	Pigment-dispersing factor	Pdfr	ACYPI46431	2	(IPR000832) GPCR, family 2, secretin-like	6.16E−49	PDF receptor	*D. melanogaster*
B5	KAF6198460.1	Pigment-dispersing factor	Pdfr	ACYPI46431	2	(IPR000832) GPCR, family 2, secretin-like	6.82E−26	PDF receptor	*D. melanogaster*
B6	KAF6210210.1	Diuretic hormone 31	hec	ACYPI009569	4	(IPR000832) GPCR, family 2, secretin-like; (IPR036445) GPCR family 2, extracellular hormone receptor domain superfamily	1.12E−52	Calcitonin receptor	*Oryctolagus cuniculus*
B7	KAF6210211.1	Diuretic hormone 31	hec	ACYPI009569	3	(IPR000832) GPCR, family 2, secretin-like	6.2E−21	Corticotropin-releasing factor receptor 1	*Mus musculus*
B8	KAF6210212.1	Diuretic hormone 31	Dh31-R	ACYPI007222, ACYPI001361	Complete	(IPR000832) GPCR, family 2, secretin-like; (IPR036445) GPCR family 2, extracellular hormone receptor domain superfamily	4.18E−84	Calcitonin gene-related peptide type 1 receptor	*Danio rerio*
B9	KAF6204739.1	Parathyroid hormone	na	na	5	(IPR000832) GPCR, family 2, secretin-like; (IPR036445) GPCR family 2, extracellular hormone receptor domain superfamily	6.86E−75	PTH2 receptor	*Homo sapiens*
**SUBFAMILY B2**									
B10	KAF6216792.1	α-latrotoxin	Cirl	ACYPI005705	Complete	(IPR000832) GPCR, family 2, secretin-like; (IPR036445) GPCR family 2, extracellular hormone receptor domain superfamily; (IPR043159) D-galactoside/L-rhamnose binding SUEL lectin domain superfamily; (IPR031234) Latrophilin-1	0	Latrophilin	*Drosophila ananassae*
B11	KAF6198557.1	Orphan	stan	ACYPI001529	Complete	(IPR000832) GPCR, family 2, secretin-like	1.59E−80	stan	*D. melanogaster*
B12	KAF6198871.1	Orphan	CG15744	na	Complete	(IPR032675) Leucine-rich repeat domain superfamily; (IPR000832) GPCR, family 2, secretin-like; (IPR013783) Immunoglobulin-like fold; (IPR036445) GPCR family 2, extracellular hormone receptor domain superfamily; (IPR036179) Immunoglobulin-like domain superfamily	2.6E−111	Adhesion GPCR A3	*Danio rerio*
**SUBFAMILY B3**									
B13	KAF6217262.1	Orphan	mthl5	ACYPI003439	Complete	None predicted	1.2E−128	Mth-like 5	*D. melanogaster*
B14	KAF6207251.1	Orphan	mthl	na	5	(IPR000832) GPCR, family 2, secretin-like	2.45E−12	Probable Mth-like 4	*D. melanogaster*
B15	KAF6215469.1	Orphan	mthl	na	Complete	(IPR000832) GPCR, family 2, secretin-like	1.91E−50	Mth2	*Drosophila simulans*
B16	KAF6197298.1	Orphan	mthl	na	Complete	(IPR000832) GPCR, family 2, secretin-like	3.21E−26	Mth2	*Drosophila yakuba*
B17	KAF6207182.1	Orphan	mthl	na	Complete	(IPR000832) GPCR, family 2, secretin-like	6.91E−21	Probable Mth-like 3	*D. melanogaster*
B18	KAF6208177.1	Orphan	mthl	na	Complete	(IPR000832) GPCR, family 2, secretin-like	4.42E−19	Probable Mth-like 4	*D. melanogaster*
B19	KAF6207243.1	Orphan	mthl	na	Complete	(IPR000832) GPCR, family 2, secretin-like	5.5E−18	Probable Mth-like 3	*D. melanogaster*
B20	KAF6216028.1	Orphan	mthl	na	Complete	(IPR000832) GPCR, family 2, secretin-like; (IPR022343) GCR1-cAMP receptor	7.79E−27	Probable Mth-like 11	*D. melanogaster*
B21	KAF6206877.1	Orphan	mthl	na	Complete	(IPR000832) GPCR, family 2, secretin-like	2.6E−33	Mth2	*Drosophila yakuba*

na, not annotated or applicable, Complete means there is a complete 7TM structure.

**Table 4:** The number of *A. lucorum* GPCRs of each family in comparison with the other four insects.

	*A. lucorum*	*A. pisum*	*D. melanogaster*	*T. castaneum*	*B. mori*
Family-A	98	62	73	68	69
Opsin	7	5	7	2	6
Biogenic amine receptors	30	18	21	20	16
Neuropeptide and protein hormone receptors (contained the purine GPCRs)	58	39	45	46	47
Family-B	21	10	26	21	12
Family-C	10	7	10	10	9
Family-F	4	3	5	4	3
Total	133	82	113	103	93

### 3.1 Family-A GPCRs

Insect family-A GPCRs include opsins, biogenic amine receptors, neuropeptide and protein hormone receptors, and purine GPCRs (17, 18, 22). In this study, 98 family-A GPCRs were identified in the genome of *A. lucorum*, and these receptors were composed of seven opsins, 30 biogenic amine receptors, 58 neuropeptide and protein hormone receptors, and three purine GPCRs (**Tables 1**, **2**).

#### 3.1.1 Opsins

Color vision in insects is based on the expression of different opsins in photoreceptive cells. Opsins are members of the family-A GPCRs and are coupled to light-sensitive chromophores in animal photoreceptors (50). Three groups of opsins have been reported in *D. melanogaster*: one related to long-wavelength (LW) vision (including Rh1, Rh2, and Rh6), another group related to short-wavelength (SW) vision (Rh3, Rh4, and Rh5), and a third group including only Rh7 (34, 51). A fourth group of invertebrate opsins, named pteropsins, has been found in *Apis mellifera* (50) and *R. prolixus* (27), which was missing from the genome of *D. melanogaster* and *A. pisum*.

**Figure 5:**
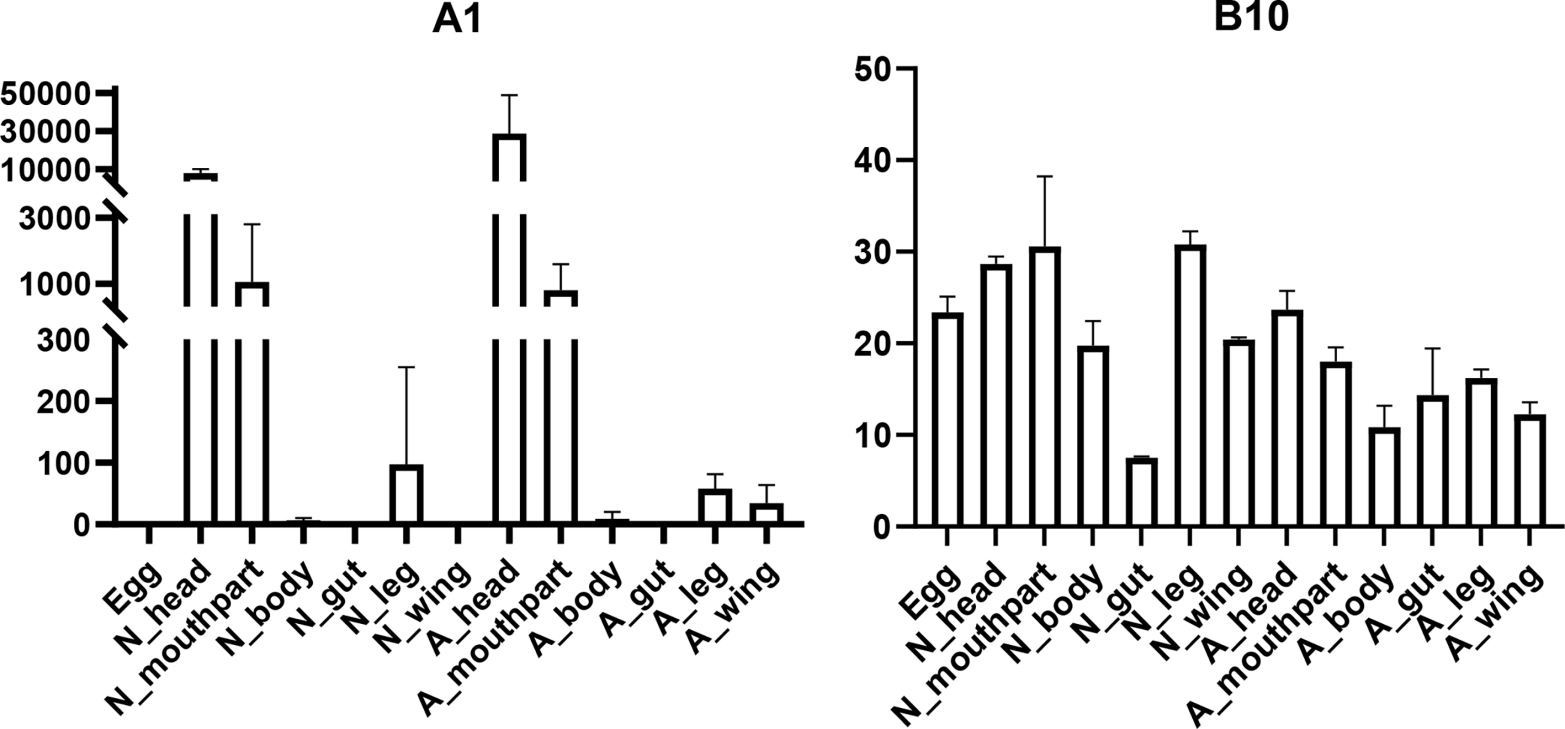
Expression patterns of A1 and B10 in different tissues by transcriptome analysis. The vertical bars indicate standard errors of the mean (n = 3).

In this study, seven putative opsins were identified in *A. lucorum*. The phylogenetic analysis suggested that A1 and A2 are related to the LW opsin, A3 is related to the SW opsin, A4 and A5 belong to a third group, and A6 and A7 are close to pteropsins (**Figure 1**). Four groups of invertebrate opsins were also identified in the *A. lucorum* genome. According to the expression profile, opsins were expressed at the highest levels in the head and mouthpart tissue, which is corresponding to their biological function. Among the four types of opsins detected in *A. lucorum*, the A1 showed the highest expression in the head tissue of adults with a transcripts per kilobase of exon model per million mapped reads (TPM) of 28,787 (**Figure 5**) followed by A3 with a TPM of 770.

#### 3.1.2 Biogenic Amine Receptors

The known biogenic amines that act as ligands for GPCRs in insects contain acetylcholine, dopamine, serotonin, octopamine, and tyramine (27). Here, we identified 30 biogenic amine receptors in *A. lucorum*. Based on phylogenetic analysis and sequence similarity, A8–11 are receptors for acetylcholine; A12–16 are dopamine-like receptors; A17–24 are orthologs of the octopamine receptors; A26–33 and A35–36 were identified as the serotonin-like receptors; and A25 is the GPCR that could be stimulated by two structurally related endogenous ligands, octopamine and tyramine (**Figure S1**). Additionally, A34 and A37 are orphan receptors of this subfamily in *A. lucorum*, and are orthologs of RPRC011175 and CG13579, respectively. However, two tyramine receptors (TyrR and TyrRII) are likely to be missing in all three heteropteran insects. A25 is the only tyramine receptor in *A. lucorum.* Compared with opsins, the expression level of biogenic amine receptors is much lower. A36 showed the highest expression in gut tissues of adults with a TPM of 11. In FlyBase (33), we found *5-HT7*, the ortholog gene of A36 in *D. melanogaster*, was also expressed in the digestive system.

#### 3.1.3 Neuropeptide and Protein Hormone Receptors

**Figure 6:**
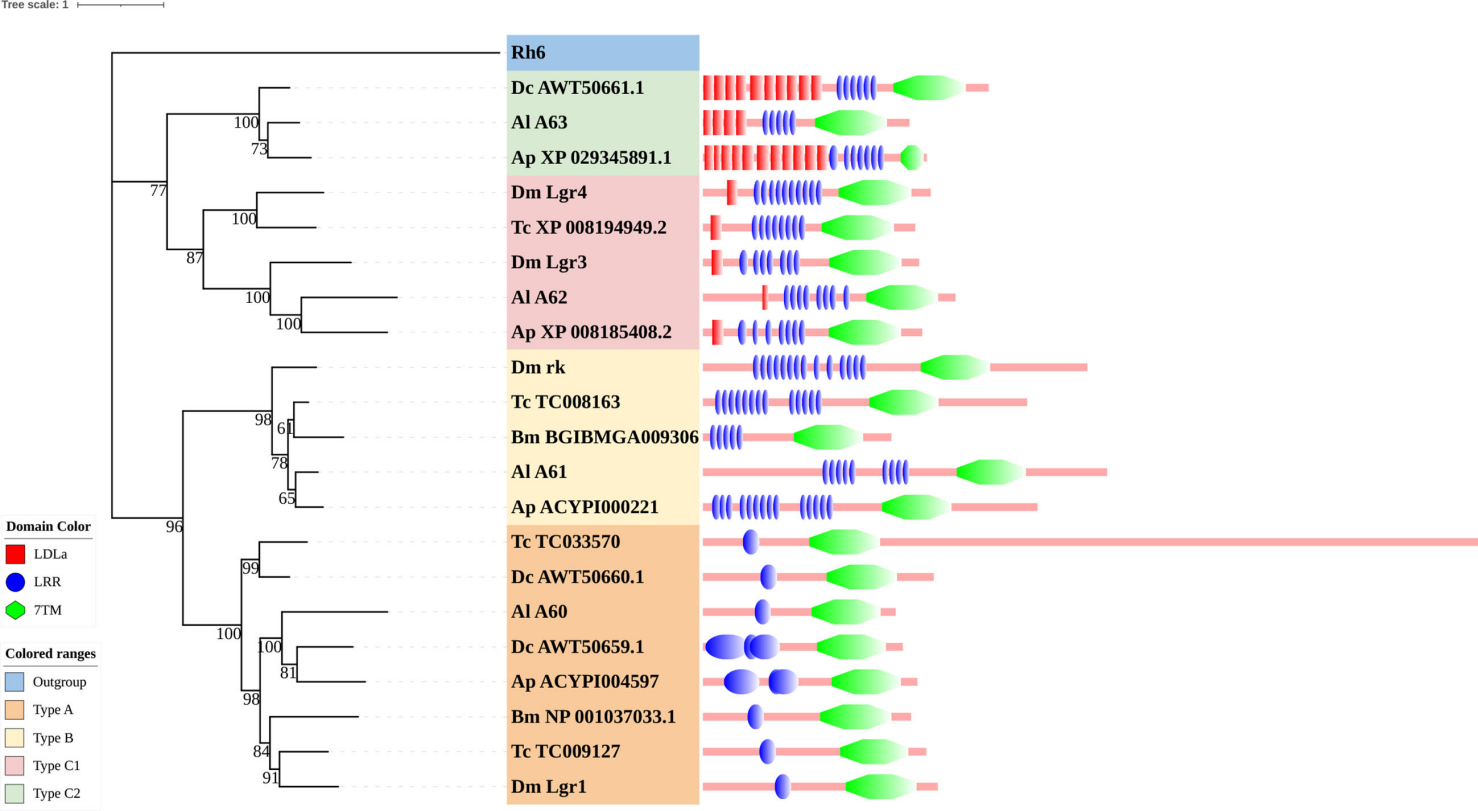
Phylogenetic tree and domains of different LGRs. Numbers at nodes on the tree were the bootstrap values (below 50 are not shown). The tree was rooted by the *D. melanogaster* opsin GPCR Rh6. Different LGR types were painted with different colors. Predicted domains of each sequence are shown in the corresponding branch side. Dc, *D*. *citri*; Al, *A. lucorum*; Ap, *A*. *pisum*; Dm, *D*. *melanogaster*; Tc, *Tribolium castaneum*; Bm, *B*. *mori*.

**Figure 7:**
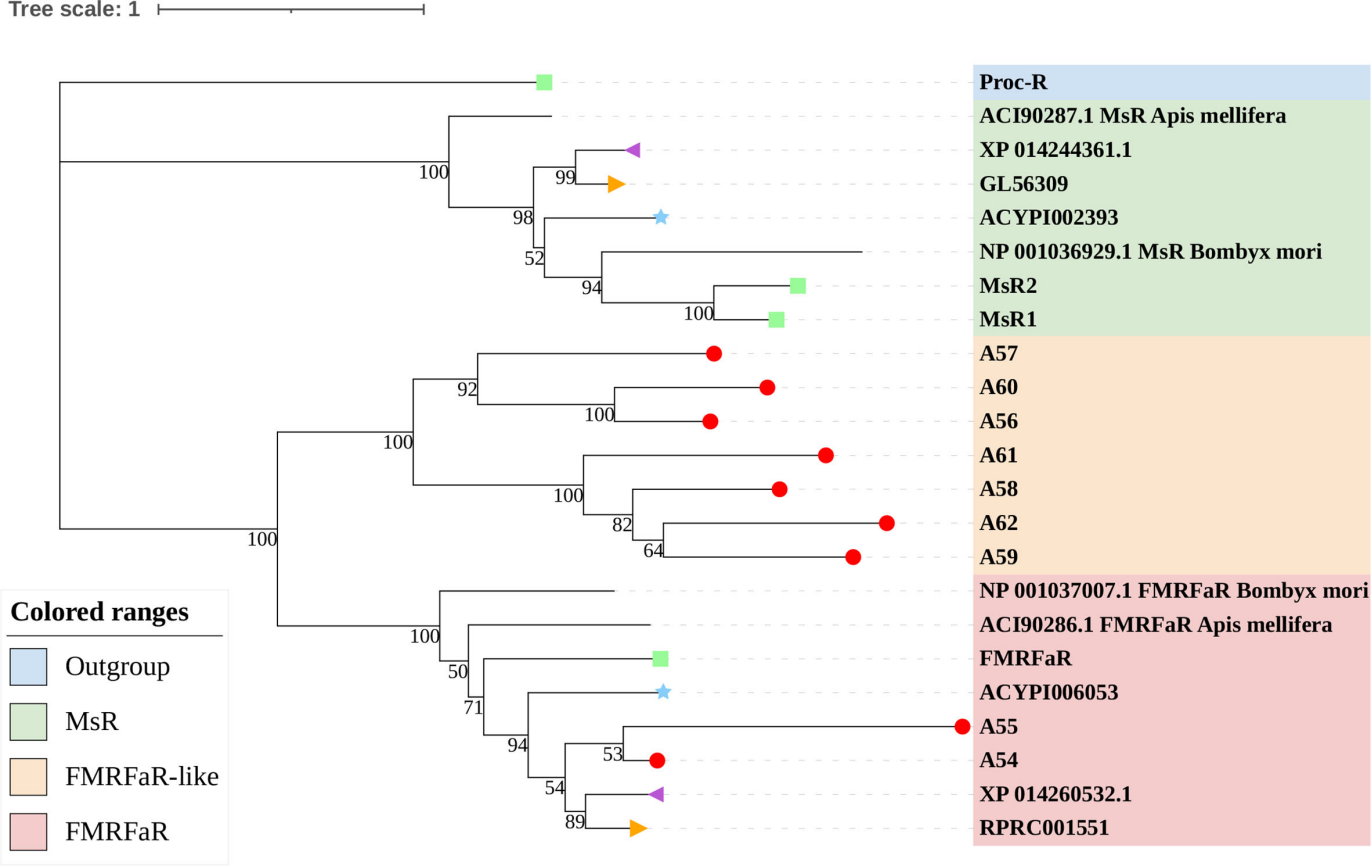
Phylogenetic tree reconstruction of FMRFaRs and MsRs from *D. melanogaster* (green square), *A. pisum* (blue star), *R. prolixus* (orange right triangle), *C. lectularius* (purple left triangle), *A. lucorum* (red circle), and other two model insects inferred from maximum likelihood (ML) analysis. Numbers at nodes on the tree were the bootstrap values. The tree was rooted by the *D. melanogaster* Proc-R.

The rhodopsin-like neuropeptide and protein hormone receptors are the largest subfamily in the rhodopsin-like family (17, 22, 52). In this subfamily, 58 putative *A. lucorum* sequences were identified. Like other insects, *A. lucorum* rhodopsin-like neuropeptide and protein hormone receptors can be classified into 25 groups based on their ligands; i.e., adipokinetic hormone receptors (AKHR), AKH/corazonin-related peptide (ACP) receptors, allatotropin receptor (AT-R), allatostatin-A receptors (AstA-R), allatostatin-B receptors (AstB-R), allatostatin-C receptors (AstC-R), bursion receptor, corazonin receptors (CrzR), neuropeptide F receptors (NPFR), short neuropeptide F receptors (sNPFR), proctolin receptors (Proc-R), pyrokinin receptors (PK-R), leukokinin receptors (Lkr), cholecystokinin-like receptors (CCKLR), tachykinin receptors (TkR), CAPA receptors (CapaR), crustacean cardioactive peptide receptors (CCAP-R), CNMamide receptors (CNMaR), CCHamide receptors (CCHa-R), ecdysis triggering hormone receptors (ETHR), FMRFamide receptors (FMRFaR), GPA2/GPB5 receptors, SIFamide receptors (SIFaR), relaxin receptors, RYamide receptors (RYa-R), and several orphan GPCRs (**Figure 2**). Most of these neuropeptide receptors displayed one-to-one orthologous relationships between *A. lucorum*, *R. prolixus*, *C. lectularius*, *A. pisum*, and *D. melanogaster*, and all subtypes of leucine-rich repeat-containing GPCRs (LGR) were observed in *A. lucorum* (**Figures 2** and **6**). However, several duplications and losses of neuropeptide receptor genes were also observed in *A. lucorum*. It is worth mentioning that as many as nine *A. lucorum* GPCRs (A54–62) displayed strong evidence of an evolutionary kinship with the FMRFaRs of *R. prolixus*, *C. lectularius*, *A. pisum*, and *D. melanogaster*, indicating that a large clade may have duplicated from FMRFaRs in *A. lucorum* (**Figures 2** and **7**). Duplications of eight neuropeptide receptor genes (*CapaR*,* CCAP-R*, *CNMaR*, *ETHR*, *Lkr*,* NPFR*,* PK1-R*, and *SIFaR*) were identified in *A. lucorum*, and duplications of *Lkr*, *PK1-R*, *ETHR*, *CCAP-R*,* NPFR*, and *SIFaR* were also observed in *R. prolixus* or *C. lectularius.* The trapped in endoderm 1 (Tre1) receptors, trissin receptors (TrissinR), myosuppressin receptors (MsR), and other six orphan receptors were not found in the genome of *A. lucorum*. Instead, we found six orphan receptors (A89–92 and A94–95) that have not been reported in *A. lucorum*. The expression levels of neuropeptide and protein hormone receptors were higher than in biogenic amine receptors. The expression of A65 (LGR) in the bodies of nymphs was the highest in this subfamily with a TPM of 41. Moreover, A50 (CCAP-R) and A86 (moody) showed high expression levels in multiple tissues (TPM >10 at least in five tissues).

#### 3.1.4 Purine GPCRs

Only one receptor in this subfamily, adenosine receptor (AdoR), has been previously classified in this subfamily (17, 53). Here, three putative *A. lucorum* GPCRs (A96–98) were identified as AdoR, whereas there was only one member in *D. melanogaster* and *A. pisum* (**Table 2**). Purine GPCRs are activated by the binding of purine nucleotides or their derivatives (principally adenosine or ADP/ATP) (54, 55). Duplication of AdoR suggests that purinergic neural transmission may play a more important role in *A. lucorum*.

### 3.2 Family-B GPCRs

Family-B GPCRs play vital roles in many biological processes, including growth, development, and reproduction. They are characterized by long N-terminal domains, and they form a small group of receptors that are structurally and functionally divergent from other groups of GPCRs (56). Within this family, family-B GPCRs can be further subdivided into three subfamilies: B1–B3, which are greatly divergent in both function and structure. In total, 21 family-B GPCRs were identified from the genome of *A. lucorum* in this study, which consisted of nine B1 subfamily members, three B2 subfamily members, and nine B3 subfamily members (**Table 3** and **Figure 3**).

The B1 subfamily is made of largely classical hormone receptors. It comprises three types of hormone receptors in *D. melanogaster*: diuretic hormone 31 receptor (DH31-R/hector), CRF-like diuretic hormone 44 (DH44-R), and pigment dispersing factor receptor (Pdfr) (57–60). In our study, all three types of hormone receptors were identified. The parathyroid hormone receptor (PTHR), which is involved in the calcium and phosphate homeostasis and bone growth in vertebrates, is also a subfamily-B1 GPCR (58). There are two PTHRs in mammals, which are involved in calcium and phosphate homeostasis and bone growth (61, 62). In insects, PTHR-like (PTHRL) have been identified from *T. castaneum*, *A. mellifera*, *P. h. humanus*, and *N. lugens* (18, 26, 57), but its counter-parts in *D. melanogaster*, *B. mori*, *A. pisum*, and *A. gambiae* are not found (17, 22, 34, 63). *T. castaneum* has two distinct PTHRLs (57), *N. lugens* possesses a pair of homologous PTHRLs (26), and *A. mellifera* only has one PTHRL (57). In our study, we also identified one PTHRL, B9, which shared a low e-value (1e−104 and 1e−111) with two PTHRLs in *N. lugens*. These results showed that genes coding for *PTHR* are divergent among insects.

The B2 subfamily is characterized by a long extracellular N-terminal domain and a GPCR proteolytic site (57, 58, 64). Based on phylogenetic analysis and sequence similarity, three receptors (B10–12) were classified in the B2 subfamily, which correspond to a calcium-independent receptor for α-latrotoxin (Cirl), starry night (stan), and CG15744, respectively. However, the orthologs for CG11318 and CG15556 were not identified in the genome of *A. lucorum*.

There is only one group of receptors in the B3 subfamily; i.e., Methuselah (mth)/Methuselah-like (mthl) (57, 58). This gene family is involved in the modulation of life span and stress responses. No counterpart for the *mth* gene family has been identified in vertebrates (57). In insects, the number in the B3 subfamily is highly variable (18, 57). In *D. melanogaster*, this subfamily can be divided into two groups based on their structure, 12 mth ectodomain‐positive members (*mth*, *mthl2*, *mthl3*, *mthl4*, *mthl6*, *mthl7*, *mthl8*, *mthl9*, *mthl10*, *mthl11*, *mthl12*, and *mthl13*) and four mth ectodomain‐negative members (*mthl1*, *mthl5*, *mthl14*, and *mthl15*) (65). In our study, nine receptors (B13–21) were identified in this family. Based on phylogenetic analysis, B13 and B20 may belong to the mth ectodomain‐positive group, and others may be members of the mth ectodomain‐negative group. At the mRNA level, B13, B18, and B21 showed a higher expression level than other mthl members, which indicated these three members of the B3 subfamily may play a more important role in *A. lucorum.*

### 3.3 Family-C GPCRs

Family-C GPCRs possess a large ligand-binding extracellular domain and form constitutive dimers (34, 66–68). There are three types of GPCRs in family-C, the glutamate and γ-amino butyric acid (GABA-B) receptors, the bride of sevenless (boss-type) receptors, and the metabotropic glutamate (mGlu) receptors. Until now, nine family-C GPCRs from *D. melanogaster* and seven from *A. pisum* have been reported. By using these reference sequences, 10 family-C members (**Table S1** nad **Figure S2**) of *A. lucorum* were identified here.

Like *A. pisum* (22), there are two GABA-B receptors (C1 and C2) in *A. lucorum*. C1 shares a 78% sequence similarity with *D. melanogaster GABA-B-R1*, while C2 has a 44% sequence identity with *D. melanogaster GABA-B-R2*. The orthologous gene to *D. melanogaster GABA-B-R3* has not been found in two Hemiptera insects. The boss-type receptor was first identified as a ligand for sevenless tyrosine kinase, which was involved in eye differentiation in *D. melanogaster*. Subsequently, *boss* has been implicated in the glucose-response (69, 70). It has been reported in *D. melanogaster* (34), *A. gambiae* (63), *A. pisum* (22), and *T. castaneum* (16), but not in *B. mori* (17), *A. mellifera*, *N. vitripennis*, and *P. humanus corporis*. Here, C3 was identified as the orthologous gene to *boss*. These results indicated that *boss* has been randomly lost in insects during their evolutionary process. Moreover, three mGlu receptors (C4, C5, and C6) were found in *A. lucorum*, whereas there are only two mGlu receptors in *D. melanogaster* (34) and one mGlu receptor in *A. pisum* (22). Small expansions of *A. lucorum* mGlu receptors have been observed. There are some unclassified receptors in this family. C7 was the orthologous gene to *smog*. C8 and C9 showed 53 and 38% sequence identities with their counterparts in *D. melanogaster*, respectively. As shown in **Figure S2**, C9 showed a high expressed level in egg, adult head, and all nymph tissues, except leg, while the function of its orthologous gene is unclear. C10 was an orphan receptor that has not been reported in *D. melanogaster* and *A. pisum* (22, 34).

### 3.4 Family-F GPCRs

Family-F GPCRs comprise the frizzled gene family and the smoothened gene (34, 71). In this study, four putative *A. lucorum* GPCRs were identified in the frizzled/smoothened GPCR family, which are orthologous to *D. melanogaster* fz, fz2, fz3, and smo (**Table S1** and **Figure S3**). Our results indicated that orthologs for *D. melanogaster* fz4 were missing in *A. lucorum*. Among family-F, F4 (smo) showed the highest expression in the egg with a TPM of 61.

## 4 Discussion

In this study, we systematically identified 133 GPCRs from *A. lucorum*. Compared with other model insects, we also found the GPCR genes remarkably expanded among the biogenic amine receptors, neuropeptide and protein hormone receptors, and the B1 subfamily (**Table 4**). Some of them had been reported in *R. prolixus* or *C. lectularius* (24, 27), such as the duplications of *5-HT7*, *Lkr*, *PK1-R*, *ETHR*, *CCAP-R*,* NPFR*, *SIFaR*, and *DH31-R*. However, missing *TyrR*, *Tre1*, *TrissinR*, *MsR*, and some orphan receptors has also been observed in the genome of *A. lucorum* (**Tables S2, S3**). All these predicted GPCRs were quantified by transcriptome data. Although most GPCR genes showed a low expression level in *A. lucorum*, there were a few highly expressed GPCR genes, such as the long-wavelength opsin and *Cirl*. By comparative analysis, we also found C2 LGR types were widely distributed in Hemiptera. All these aspects will be discussed in detail below.

### 4.1 GPCRs Gene Expansion Occurred in *A. lucorum*

Compared with other well-studied insects, we noticed that the number of genes coding for GPCRs is obviously larger than for other insects, especially expanded among the biogenic amine receptors, neuropeptide and protein hormone receptors, and B1 subfamily (**Table 4**). There were 26 GPCRs duplicated in *A. lucorum*. Twenty-three of them were classified into the three subfamilies mentioned above. By MCScanX analysis, we found six tandem duplication events occurred among Rh6, Rh7, AstA-R, ETHR, FMRFaR, and AdoR, while most GPCR genes duplicated dispersedly. Considering the location of GPCR genes on chromosomes, it suggested that the duplication of GPCR genes mainly occurred as independent duplications and transitions (**Figure 4**).

The duplicate biogenic amine receptors in *A. lucorum* included 5-HT1A, 5-HT1B, 5-HT2A, 5-HT2B, 5-HT7, Dop2R, mAChR-B, Oamb, Octbeta2R, and Octbeta3R. These biogenic amine receptors can regulate many behaviors including flight and fight, learning and memory, sleep and wakefulness, feeding, and social and reproductive behaviors (72–74). For example, 5-HT1A was related to locomotor activity in *B. mori*. Injecting the antagonist of the Bm5-HT1A receptor into larvae caused slow or weak motility, and adults had lowered courtship vitality or moving speed (75). mAChRs have also been reported to be critical in regulation of locomotory behavior in *Drosophila* (76). In addition, 5-HT1B mediates hemocyte phagocytosis and serotonergic signaling performs critical modulatory functions in immune systems. Moreover, 5-HT7 and Dop2R were shown to be associated with learning ability (77, 78) and octopamine receptors were required for ovulation in *D. melanogaster* (79). The ligands of neuropeptide and protein hormone receptors and the B1 subfamily belong to neuropeptides, which also play an important role in the regulation of development, reproduction, feeding, courtship, aggression, olfaction, locomotor activity, circadian rhythm, and many other physiological processes in insects (21, 80, 81). The gene expansion of these three subfamilies indicated that *A. lucorum* had a more complex peptidergic signaling system.

Expansions of genes associated with omnivorousness and mesophyll feeding, such as those related to digestion, chemosensory perception, and detoxification, were also observed in *A. lucorum* (32). Gustatory receptors (Grs) and odorant receptors (Ors) are thought to be the most important chemosensory receptors. Like GPCRs, Ors, and Grs are seven-transmembrane domain receptors but belong to the chemosensory 7tm receptor superfamily (82, 83). It has been suggested that the cause of gene expansion in GPCRs might be similar to that of chemosensory receptors, also to better adapt to the environment (32). *A. lucorum* is found in natural and agricultural ecosystems throughout the world (30), and many of them are generalists, exhibiting diverse feeding habits or preferences (e.g., feeding on leaf, stem, inflorescences, nectar, pollen, and fruit) (32). These results indicated the complex peptidergic signaling system is more favorable for *A. lucorum* to adapt to multiple living environments and multiple hosts.

### 4.2 *A. lucorum* Appears to Have Evolved From a Novel Large Clade of Known FMRFaRs

FMRFamide (FMRFa) is a cardioexcitatory peptide that was first isolated from the nervous system of the clam, *Macrocallista nimbosa* (84), and is active as a tetrapeptide only in mollusks and annelids. Since the discovery of FMRFa, peptides with extended length at the N-terminal portion have been reported, such as myosuppressin (Ms) (85). Here, nine receptors (A54–62) displayed a certainly evolutionary kinship with the FMRFaR of *D. melanogaster*, *A. pisum*, *C. lectularius*, and *R. prolixus*, while the MsR is missing in *A. lucorum* (**Figure 2**). By reconstruction of the phylogenetic tree of MsR and FMRFaR with more species (17, 86), we found these receptors were closer to the known FMRFaRs (**Figure 7**). Among these, A54 and A55 were clustered in a single clade with the FMRFaRs that had been identified in other insects, whereas the other seven receptors are clustered on the other single clade. The most similar proteins in UniProtKB/Swiss-Prot of these receptors are both *D. melanogaster* FMRFaRs (CG2114). Among these receptors, A54 was the ortholog to the insect FMRFaRs with the smallest e-value of 1.75E−130, and A55, which was adjacent located on chromosome 9, is a tandem duplication that occurred at the beginning of this receptor expansion. However, the other seven receptors (A56–62) were scattered across six chromosomes, which indicated these seven receptors might have arisen from transposition.

By searching in the genome of other heteropteran insects, we found there are only one or two FMRFaRs in each heteropteran species. We suggest A54 and A55 should be classified as FMRFaRs, while the others (A56–62) were named as FMRFaR-like for the moment. This branch might be another unknown GPCR or even contain the MsR. Recent research had found that FMRFaR stimulates intracellular calcium signaling through the IP3R and helps maintain neuronal excitability in a subset of dopaminergic neurons for positive modulation of flight bout durations (87), and the ligand can reduce spontaneous muscle contractions, such as in the intestinal muscle and the heart rate, which also have an impact on movements (88, 89). *A. lucorum* has great flight capacity and its adults can fly 151.3 km within 48 h (90). A large FMRFaR-like branch evolved in *A. lucorum* may help it maintain strong flight capability.

### 4.3 Only a Few GPCR Genes Showed High Expression Levels in *A. lucorum*

In our research, only 23 GPCR genes (17.3% of all GPCR genes) were expressed highly (TPM >10) in at least one tissue, while most GPCR genes showed a low expression level in *A. lucorum*. This result is consistent with previous studies in which most GPCRs showed a low endogenous expression level, even at the mRNA level (91–93). Certainly, a low expression level of GPCRs does not necessarily equate to functional insignificance (94).

Here, through transcriptome analysis, we found the expression levels of opsins were higher than in other subfamilies. Opsins that originated early in metazoan evolution mediate the response to visual stimuli primarily. When stimulated by light, opsins can activate a downstream signaling cascade by conformational change (95). Most opsin genes are expressed in photoreceptors, but there are opsins expressed in other tissues, suggesting some nonvisual functions (96, 97). In *A. lucorum*, opsin genes (A1–3) were expressed highly not only in head but also in leg, wing, and mouthpart, indicating these opsins may execute some nonvisual functions (**Figure 1**). Among these, the LW opsin (A1) showed the highest expression levels in the head tissue of adults (TPM = 28,787, at least 20 times more than other GPCR; **Figure 5**). The peak absorbance of the LW opsin is 500–600 nm, which corresponds to yellow-green light. As night sets in, the natural ambient light is increasingly dominated by longer wavelengths (98, 99). The importance of LW opsin had been reported in many nocturnal insects (100, 101). The adults of the *A. lucorum* were mainly active from dusk to early morning (102). High expression levels of LW opsin may help the organism adapt to a low light environment. We found B10, orthologous to Cirl, is the most widely expressed GPCR gene, which can be tested in all tissues (**Figure 5**). Cirl belongs to a unique branch of GPCRs and, specifically, is an adhesion GPCR (103, 104). The orthologs of Cirl have been discovered in almost all animals from invertebrates to vertebrates, including humans (105). There are three homologs of Cirl in most vertebrates (Cirl‐1, Cirl‐2, and Cirl‐3) and two in birds and worms, whereas there is only one homolog in insects—which is most homologous to vertebrate Cirl-2 (103, 106). The expression pattern of insects *Cirl*, which had been reported to be expressed in multiple tissues (103, 107), was also like vertebrate *Cirl-2* (108). Although there is only one *Cirl* member in insect species, *Cirl* is still involved in multiple physiological processes, which can regulate sensory, developmental, reproductive, and immune functions in insects (104, 109). Here, B10 had been detected in all transcriptomic samples and its distribution range is wider than in *T. castaneum* and *D. melanogaster* (103, 106). This kind of expression pattern suggested B10 is crucial in the development of *A. lucorum*.

### 4.4 Type C2 LGRs Are Mainly Distributed in Hemiptera and Phthiraptera Insects

Within the neuropeptide and protein hormone receptor subfamily, LGRs are a distinct subgroup with important functions in development and reproduction (110). Three distinct types of LGRs have been defined based on their structural characteristics and they are distinguished by the number of leucine-rich repeat (LRR) motifs, the absence or presence of a low density lipoprotein receptor domain class A (LDLa) motif, and their type-specific hinge region. Generally, type B LGRs have about twice the number of LRRs compared to the other two types. An exclusive feature of the type C LGRs is the presence of at least one LDLa motif in the ectodomain. The more general type containing only one LDLa will be referred to as type C1, whereas type C2 contained more than one LDLa (111).

Type C2 LGRs were first discovered in echinoderms, mollusks, and in one insect species (*Pediculus humanis corporis*). In our study, we found it existed in all hemipteran insects that we studied. Combining recent work, we mentioned that type C2 LGRs are reported in many hemipteran insects and *P. h. humanus* (18, 23, 25, 26), and are lost in other orders of insects (16, 17, 63, 86) (**Figure 6**). Until now, the presence of type C2 LGRs have been found in all Hemiptera insects in which their GPCRs have been identified (22, 23, 25, 26). Among insects, Phthiraptera is one of the orders most closely related to Hemiptera (112). Type C2 LGRs may be present in the common ancestor of these two orders. To clarify the distribution of type C2 LGRs in insects, we checked all protein sequences in the non-redundant protein sequences database (nr) of NCBI. The result showed, except for Hemiptera and Phthiraptera, type C2 LGRs were also founded in *Zootermopsis nevadensis* of Blattodea. In terms of functionality, LGRs have important functions in development and reproduction. Type A LGRs and type B LGRs are stimulated by large dimeric protein hormones (110), regulating the adult eclosion of insects, and cuticle tanning (113, 114), while type C1 LGRs are the receptors of insulin-like peptide 7 and insulin-like peptide 8 and they coordinate organ growth in *D. melanogaster* (115–117). At present, the function of type C2 LGRs is undetermined. The function of type C2 LGRs and the existence of type C2 LGRs in Phthiraptera need to be explored in future work.

## Data Availability Statement

The original contributions presented in the study are included in the article/**Supplementary Material**. Further inquiries can be directed to the corresponding author.

## Author Contributions

The majority of the work described here was carried out by HG. This work was also assisted by YL, MW, XS, JT, and FF. BL designed the study and crucially revised the manuscript for important intellectual content and data analysis. All authors contributed to the article and approved the submitted version.

## Funding

This work was supported by the National Natural Science Foundation of China (Nos. 31872970 & 3217030192), and the Graduate Research and Innovation Projects of Jiangsu Province (No. KYCX21_1361).

## Conflict of Interest

The authors declare that the research was conducted in the absence of any commercial or financial relationships that could be construed as a potential conflict of interest.

## Publisher’s Note

All claims expressed in this article are solely those of the authors and do not necessarily represent those of their affiliated organizations, or those of the publisher, the editors and the reviewers. Any product that may be evaluated in this article, or claim that may be made by its manufacturer, is not guaranteed or endorsed by the publisher.
