# Supplementary material for: Identification and Expression Analysis of G Protein-Coupled Receptors in the Miridae Insect Apolygus lucorum
Source: Front Endocrinol (Lausanne). 2021 Nov 26;12:773669. doi: 10.3389/fendo.2021.773669 (PMC8660763; doi:10.3389/fendo.2021.773669)
Supplement: Supplementary file 1 [file DataSheet_1.docx]

Supplementary Material

# Supplementary Figures and Tables

##
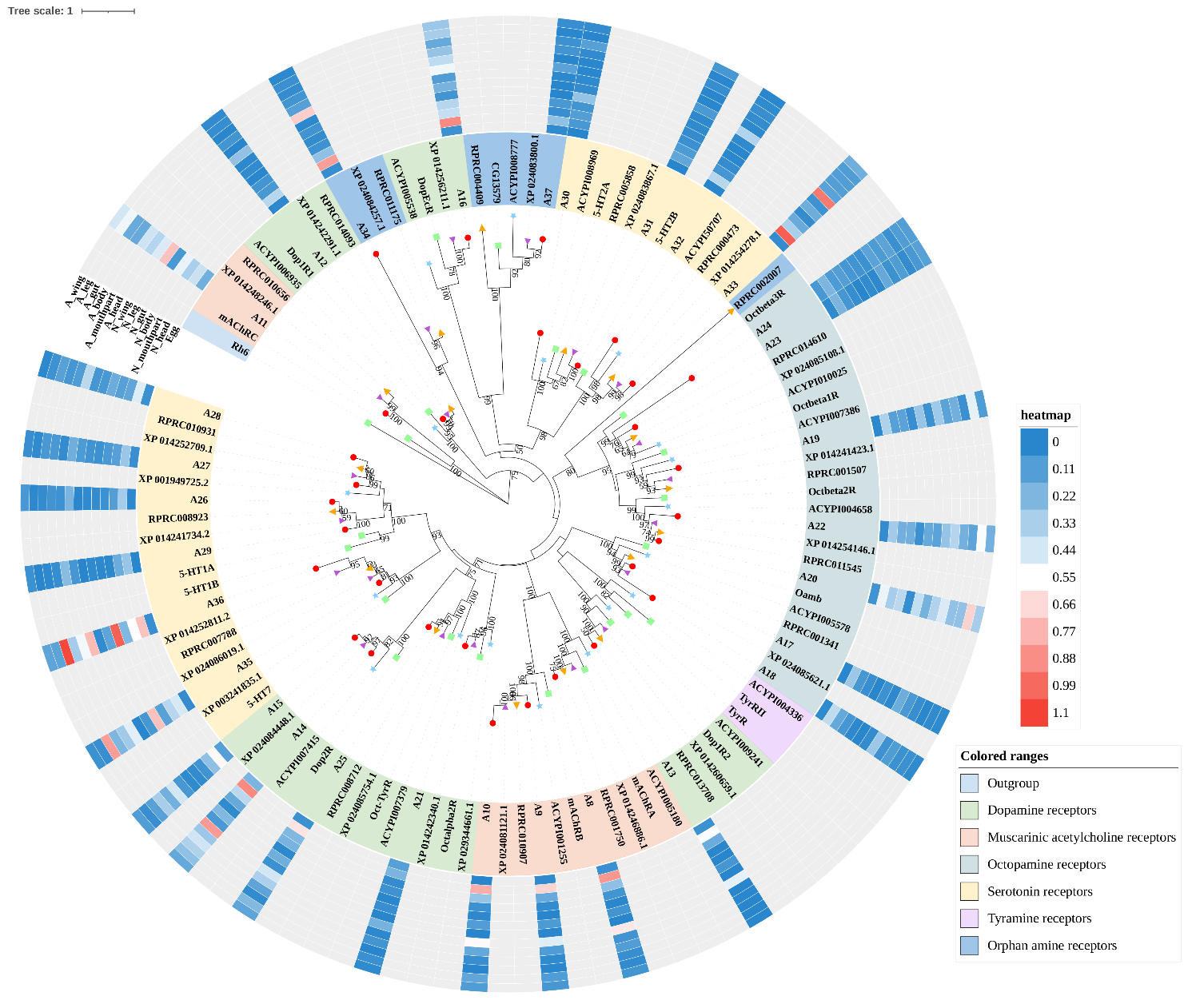
Supplementary Figures

**Fig S1.** Phylogenetic tree reconstruction of biogenic amine receptors from *D. melanogaster* (green square), *A. pisum* (blue star), *R. prolixus* (orange right triangle), *C. lectularius* (purple left triangle) and *A. lucorum* (red circle) inferred from maximum likelihood (ML). Numbers at nodes on the tree were the bootstrap values (below 50 are not shown). The tree was rooted by the *D. melanogaster* opsin GPCR Rh6. Expression profiles of *A. lucorum* GPCR genes from different tissues are shown in the corresponding branch side. The transcription level of each gene is represented by a square with a color that codes for the values of Lg (TPM+1). N, nymph; A, adult.

**
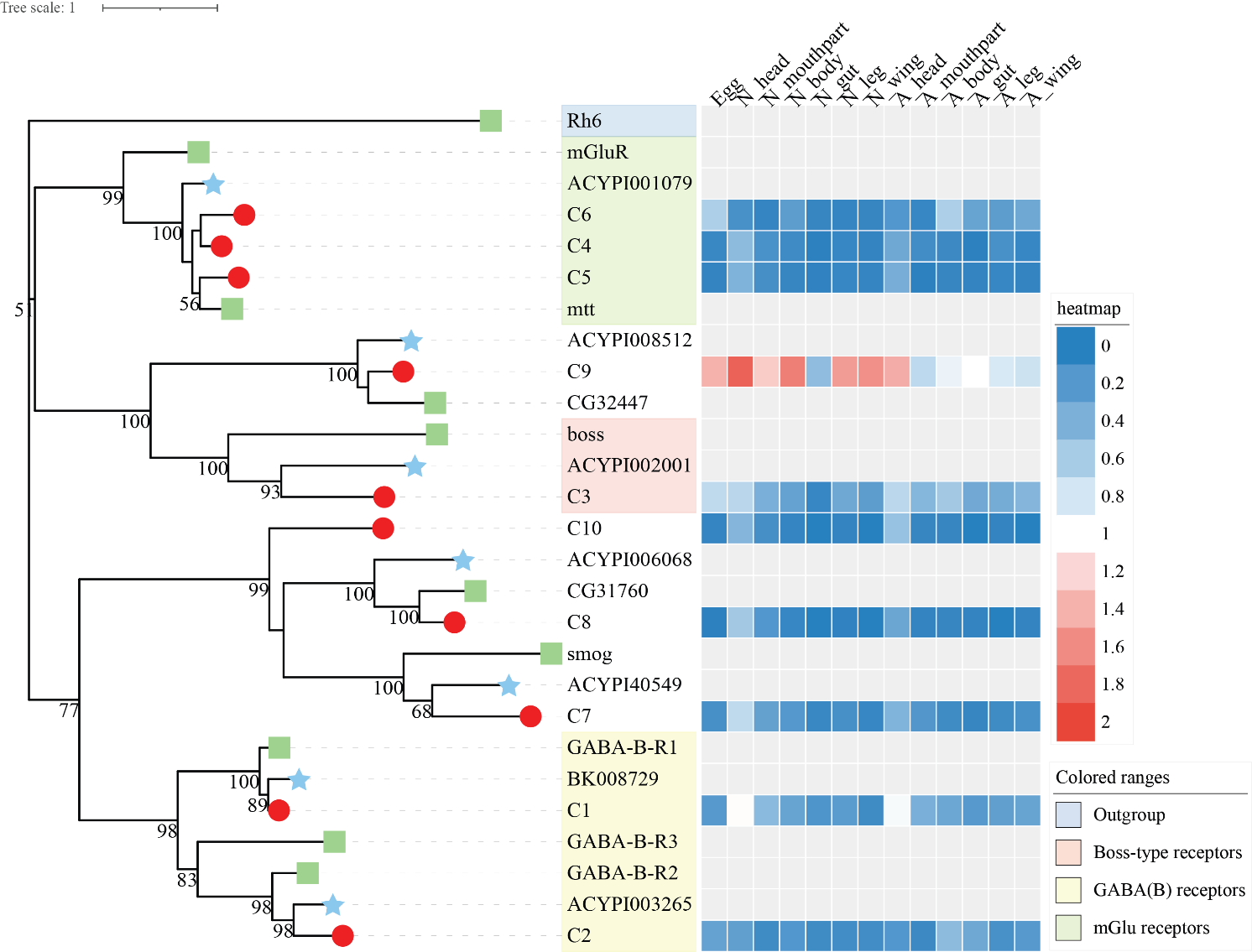
**

**Fig S2.** Phylogenetic tree reconstruction of Family-C GPCRs from *D. melanogaster* (green square), *A. pisum* (blue star), *R. prolixus* (orange right triangle), *C. lectularius* (purple left triangle) and *A. lucorum* (red circle) inferred from maximum likelihood (ML). Numbers at nodes on the tree were the bootstrap values (below 50 are not shown). The tree was rooted by the *D. melanogaster* opsin GPCR Rh6. Expression profiles of *A. lucorum* Family-C GPCR genes from different tissues are shown in the corresponding branch side. The transcription level of each gene is represented by a square with a color that codes for the values of Lg (TPM+1). N, nymph; A, adult.

**
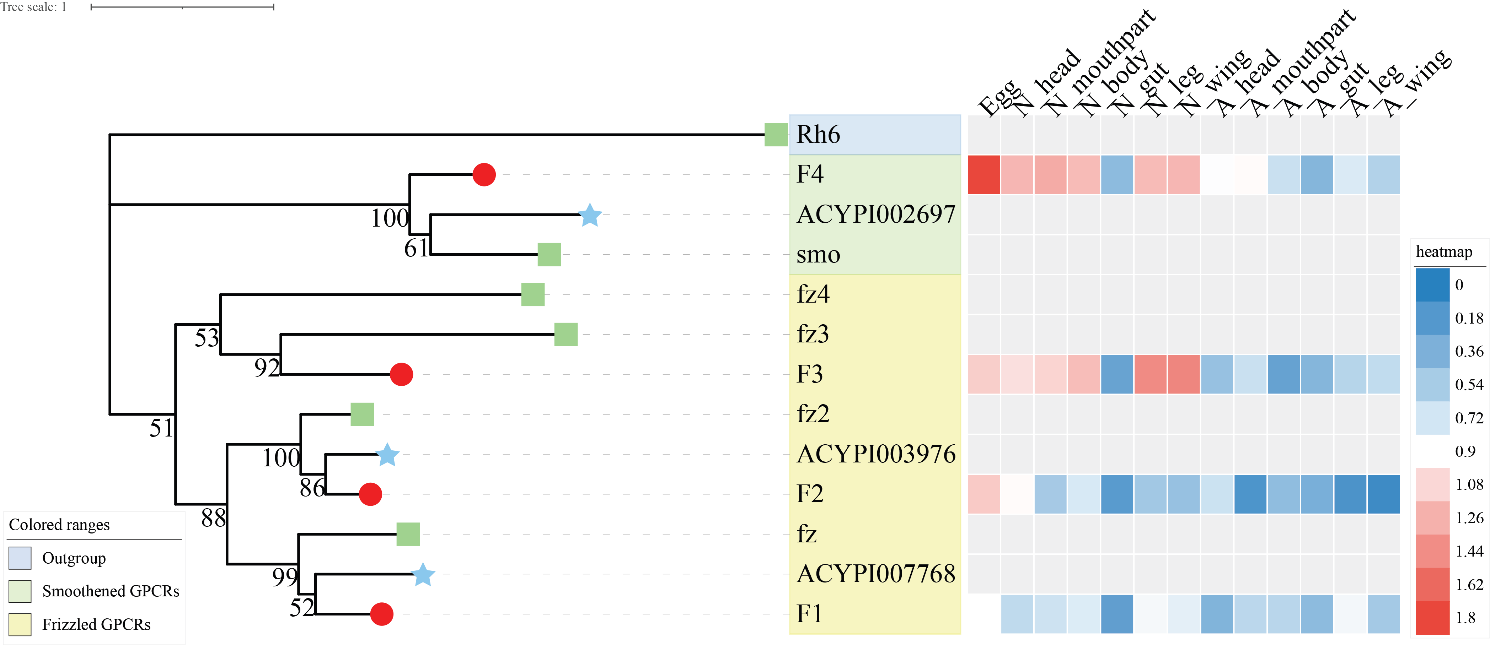
**

**Fig S3.** Phylogenetic tree reconstruction of Family-F GPCRs from *D. melanogaster* (green square), *A. pisum* (blue star), *R. prolixus* (orange right triangle), *C. lectularius* (purple left triangle) and *A. lucorum* (red circle) inferred from maximum likelihood (ML). Numbers at nodes on the tree were the bootstrap values (below 50 are not shown). The tree was rooted by the *D. melanogaster* opsin GPCR Rh6. Expression profiles of *A. lucorum* Family-F GPCR genes from different tissues are shown in the corresponding branch side. The transcription level of each gene is represented by a square with a color that codes for the values of Lg (TPM+1). N, nymph; A, adult.

## Supplementary Tables

**Table S1.** Family-C and Family-F GPCRs of *A. lucorum*

| No. | Accession number | Putative Endogenous ligand | orthologue of *D. melanogaster* | orthologue of *A. pisum* | predicted TMHs | Annotation by InterProScan | Homology search in Swissport (blastp) | | |
| --- | --- | --- | --- | --- | --- | --- | --- | --- | --- |
|  |  |  |  |  |  |  | E-value | description | species |
| **Class C: Metabotropic glutamate receptor-like** | | | |  |  |  |  |  |  |
| C1 | KAF6204850.1 | metabotropic GABA-B | GABA-B-R1 | BK008729 | Complete | (IPR002455) GPCR family 3, GABA-B receptor; (IPR028082) Periplasmic binding protein-like I | 0 | GABA-B receptor 1 | *Mus musculus* |
| C2 | KAF6202271.1 | metabotropic GABA-B | GABA-B-R2 | ACYPI003265 | 6 | (IPR002455) GPCR family 3, GABA-B receptor | 8.58E-118 | GABA-B receptor 2 | *Homo sapiens* |
| C3 | KAF6208749.1 | Orphan | boss | ACYPI002001 | Complete | (IPR028082) Periplasmic binding protein-like I; (IPR002956) Bride of sevenless protein | 3.58E-47 | boss | *D. melanogaster* |
| C4 | KAF6209757.1 | Orphan | mGlu | ACYPI001079 | Complete | (IPR000832) GPCR, family 2, secretin-like; (IPR036445) GPCR family 2, extracellular hormone receptor domain superfamily | 0 | mGluR8 | *Mus musculus* |
| C5 | KAF6210444.1 | Orphan | mGlu | ACYPI001079 | Complete | (IPR000337) GPCR, family 3; (IPR000162) GPCR, family 3, metabotropic glutamate receptor;(IPR038550) GPCR, family 3, nine cysteines domain superfamily; (IPR028082) Periplasmic binding protein-like I | 0 | mGluR8 | *Homo sapiens* |
| C6 | KAF6209121.1 | Orphan | mtt | ACYPI001079 | Complete | (IPR000162) GPCR, family 3, metabotropic glutamate receptor; (IPR038550) GPCR, family 3, nine cysteines domain superfamily; (IPR028082) Periplasmic binding protein-like I | 9.35E-128 | mGluR7 | *Homo sapiens* |
| C7 | KAF6214717.1 | Orphan | smog | ACYPI40549 | Complete | (IPR043458) GPCR 158/179 | 6.02E-68 | Probable GPCR 158 | *Bos taurus* |
| C8 | KAF6213839.1 | Orphan | CG31760 | ACYPI006068 | Complete | (IPR009030) Growth factor receptor cysteine-rich domain superfamily | 0 | Probable CG31760 | *D. melanogaster* |
| C9 | KAF6204363.1 | Orphan | CG32447 | ACYPI008512 | Complete | None predicted | 2.29E-11 | mGluR6 | *Homo sapiens* |
| C10 | KAF6216618.1 | Orphan | na | ACYPI006068 | Complete | (IPR000337) GPCR, family 3; (IPR043458) GPCR 158/179 | 3.53E-74 | Probable CG31760 | *D. melanogaster* |
| **Class F: Frizzled/Smoothened receptor** | | |  |  |  |  |  |  |  |
| F1 | KAF6215513.1 | Orphan | fz | ACYPI007768 | 6 | (IPR026552) Frizzled-7; (IPR015526) Frizzled/secreted frizzled-related protein; (IPR036790) Frizzled cysteine-rich domain superfamily | 0 | Frizzled-7-A | *Xenopus laevis* |
| F2 | KAF6203400.1 | Orphan | fz2 | ACYPI003976 | Complete | (IPR036790) Frizzled cysteine-rich domain superfamily; (IPR015526) Frizzled/secreted frizzled-related protein | 0 | Frizzled-2 | *D. melanogaster* |
| F3 | KAF6213886.1 | Orphan | fz3 | na | Complete | (IPR036790) Frizzled cysteine-rich domain superfamily; (IPR015526) Frizzled/secreted frizzled-related protein | 5.1E-127 | Frizzled-10-A | *Xenopus laevis* |
| F4 | KAF6213325.1 | Orphan | smo | ACYPI002697 | Complete | (IPR036790) Frizzled cysteine-rich domain superfamily; (IPR026544) Smoothened; (IPR015526) Frizzled/secreted frizzled-related protein | 0 | Protein smoothened | *D. melanogaster* |

na, not annotated or applicable, Complete means there is a complete 7TM structure.

**Table S2.** Number of biogenic amine receptors in different Hemipteran species

|  | *A. lucorum* | *R. prolixus*^[1]^ | *C. lectularius*^[2]^ | *A. pisum*^[3]^ |
| --- | --- | --- | --- | --- |
| Dopamine receptors | 7 | 3 | 6 | 6 |
| Muscarinic acetylcholine receptors | 4 | 3 | 3 | 2 |
| Serotonin receptors | 10 | 5 | 6 | 4 |
| Octopamine receptors | 7 | 4 | 4 | 4 |
| Tyramine receptors | 0 | 0 | 0 | 1 |
| Total | 28 | 15 | 19 | 17 |

[1] Ons S, *et al*. Identification of G protein coupled receptors for opsines and neurohormones in *Rhodnius prolixus*. Genomic and transcriptomic analysis. *Insect Biochemistry and Molecular Biology* (2016)

[2] Benoit JB, *et al*. Unique features of a global human ectoparasite identified through sequencing of the bed bug genome. *Nat Commun* (2016) 7:10165.

[3] Li C, *et a*l. Identification of G protein-coupled receptors in the pea aphid, *Acyrthosiphon pisum*. *Genomics* (2013) 102:345–354.

The number of orphan receptors was excluded in comparison.

**Table S3.** Number of biogenic amine receptors in different Hemipteran species

|  | *A. lucorum* | *R. prolixus*^[1]^ | *C. lectularius*^[2]^ | *A. pisum*^[3]^ |
| --- | --- | --- | --- | --- |
| AKH/ACP/Crz receptor | 3 | 2 | 3 | 1 |
| Ast-R | 4 | 4 | 4 | 3 |
| AT-R | 1 | 0 | 1 | 0 |
| CapaR | 2 | 0 | 1 | 1 |
| CCAP-R | 3 | 2 | 2 | 1 |
| CCHa-R | 1 | 2 | 2 | 3 |
| CCKLR | 1 | 3 | 1 | 0 |
| CNMaR | 2 | 1 | 1 | 0 |
| DH-R | 6 | 7 | 5 | 4 |
| ETHR | 2 | 2 | 1 | 1 |
| FMRFaR | 2* | 1 | 1 | 1 |
| LGR | 4 | 4 | 4 | 4 |
| Lkr | 2 | 2 | 2 | 2 |
| moody | 1 | 0 | 1 | 1 |
| MsR | 0 | 1 | 1 | 1 |
| NPFR | 3 | 2 | 2 | 1 |
| Pdfr | 2 | 1 | 1 | 1 |
| PK-R | 3 | 1 | 3 | 2 |
| Proc-R | 1 | 1 | 1 | 1 |
| Rya-R | 1 | 0 | 1 | 2 |
| SIFaR | 2 | 2 | 2 | 2 |
| sNPF-R | 1 | 2 | 1 | 1 |
| TkR | 2 | 2 | 3 | 2 |
| Tre | 0 | 0 | 0 | 0 |
| TrissinR | 0 | 0 | 0 | 0 |
| Total | 49* | 42 | 44 | 35 |

[1] Ons S, *et al*. Identification of G protein coupled receptors for opsines and neurohormones in *Rhodnius prolixus*. Genomic and transcriptomic analysis. *Insect Biochemistry and Molecular Biology* (2016)

[2] Benoit JB, *et al*. Unique features of a global human ectoparasite identified through sequencing of the bed bug genome. *Nat Commun* (2016) 7:10165.

[3] Li C, *et a*l. Identification of G protein-coupled receptors in the pea aphid, *Acyrthosiphon pisum*. *Genomics* (2013) 102:345–354.

* refers to the FMRFaR-like that may exist in *A. pisum*. The number of orphan receptors was excluded in comparison.
